# Comprehensive computational analysis of epigenetic descriptors affecting CRISPR-Cas9 off-target activity

**DOI:** 10.1186/s12864-022-09012-7

**Published:** 2022-12-06

**Authors:** Jeffrey K. Mak, Florian Störtz, Peter Minary

**Affiliations:** Department of Computer Science, University of Oxford, Parks Road, OX1 3QD Oxford, UK

**Keywords:** CRISPR-Cas9, Off-target activity, Nucleosome, crisprSQL, Gene editing, Machine Learning

## Abstract

**Background:**

A common issue in CRISPR-Cas9 genome editing is off-target activity, which prevents the widespread use of CRISPR-Cas9 in medical applications. Among other factors, primary chromatin structure and epigenetics may influence off-target activity.

**Methods:**

In this work, we utilize crisprSQL, an off-target database, to analyze the effect of 19 epigenetic descriptors on CRISPR-Cas9 off-target activity. Termed as 19 epigenetic features/scores, they consist of 6 experimental epigenetic and 13 computed nucleosome organization-related features. In terms of novel features, 15 of the epigenetic scores are newly considered. The 15 newly considered scores consist of 13 freshly computed nucleosome occupancy/positioning scores and 2 experimental features (MNase and DRIP). The other 4 existing scores are experimental features (CTCF, DNase I, H3K4me3, RRBS) commonly used in deep learning models for off-target activity prediction. For data curation, MNase was aggregated from existing experimental nucleosome occupancy data. Based on the sequence context information available in crisprSQL, we also computed nucleosome occupancy/positioning scores for off-target sites.

**Results:**

To investigate the relationship between the 19 epigenetic features and off-target activity, we first conducted Spearman and Pearson correlation analysis. Such analysis shows that some computed scores derived from training-based models and training-free algorithms outperform all experimental epigenetic features. Next, we evaluated the contribution of all epigenetic features in two successful machine/deep learning models which predict off-target activity. We found that some computed scores, unlike all 6 experimental features, significantly contribute to the predictions of both models. As a practical research contribution, we make the off-target dataset containing all 19 epigenetic features available to the research community.

**Conclusions:**

Our comprehensive computational analysis helps the CRISPR-Cas9 community better understand the relationship between epigenetic features and CRISPR-Cas9 off-target activity.

**Supplementary Information:**

The online version contains supplementary material available at 10.1186/s12864-022-09012-7.

## Background

CRISPR-Cas9 systems are powerful tools for site-directed binding and mutagenesis across a wide variety of eukaryotic species [1–7]. The single guide RNA (sgRNA) in CRISPR-Cas9 is highly programmable and easy to design. As a result, CRISPR-Cas9 has seen its use in many applications. Such applications include targeted genome editing, modulation of gene expression [8–10], chromatin visualization [11, 12], epigenetic modifications [13, 14], and chromatin reorganization [15]. Notably, *Streptococcus pyogenes* Cas9 is frequently used due to its short 5’-NGG-3’ PAM sequence, which is commonly found in GC-rich mammalian genomes. Nonetheless, CRISPR-Cas9 systems are currently not widely adopted in medical applications, since potential off-target Cas9 endonuclease activity [16–18] may result in undesirable biological effects [19]. To better understand off-target activity, various studies have sought to determine the different factors which influence off-target activity.

A potentially important factor which affects off-target activity is the hierarchical chromatin structure which may block off certain genomic regions. Specifically, previous experimental studies reported less CRISPR-Cas9 cleavage for target sites in heterochromatin compared to those in euchromatin in Cas9 mutagenesis experiments [20, 21]. A similar phenomenon with CRISPR-Cas9 binding activity is observed in dCas9 binding experiments [22–24]. Similarly, chromatin accessibility was observed to positively correlate with CRISPR-Cas9 activity [25, 26]. Chromatin state can be inferred by experimental epigenetic features such as DNase I hypersensitivity, CpG methylation and histone marks. These three features can be experimentally measured by DNase-seq [27], reduced representation bisulfite sequencing (RRBS) [28, 29] and histone ChIP-seq screens [30]. Because of this, various biological studies have used these experimental techniques for investigating the impact of the three epigenetic features (or scores) on off-target activity [31]. In particular, DNase I hypersensitivity and CpG methylation were observed to be highly indicative of dCas9 off-target activity [32]. However, CpG methylation was shown to indirectly contribute to off-target activity. This is because it is the DNA-binding methylation-associated factors which likely block Cas9 binding, rather than CpG methylation [20].

On the computational side, recent deep learning-based CRISPR-Cas9 off-target activity prediction tools [33–35] have used epigenetic features to represent the chromatin state at off-target sites. Such features include CCCTC-binding factor (CTCF, [36]), chromatin immunoprecipitation (ChIP, [37]), histone-3 lysine-4 trimethylation (H3K4me3, [38]), reduced representation bisulfite sequencing (RRBS, [28, 29]) and Deoxyribonuclease-I hypersensitive sites sequencing (DNase-seq, [27]) assays. Available in crisprSQL [39], DNA:RNA ImmunoPrecipitation and high-throughput sequencing (DRIP) is an epigenetic score which measures R-loop formation in the genome  [40, 41]. Notably, R-loops play a role in regulating chromatin states [42].

Alternatively, local chromatin structure can be defined as the nucleosome organization at the local region. Nucleosome organization can be described by nucleosome occupancy or nucleosome positioning. Nucleosome occupancy is defined as the cell and time-averaged probability that a given base pair participates in the nucleosomal DNA wrapping around any histone octamer. Nucleosome positioning is defined as the cell and time-averaged probability that a given base pair sits at the center of any 147bp nucleosomal DNA [43]. Nucleosome occupancy is typically measured by Micrococcal Nuclease digestion with deep sequencing (MNase-seq) [44, 45]. Various studies demonstrate that nucleosomes directly inhibit Cas9 binding and cleavage both *in vitro* and *in vivo* [23, 31, 46, 47]. However, access to nucleosomal DNA can be partially recovered via chromatin remodelling [23] and spontaneous nucleosome breathing [47].

In light of the above, we aim to conduct a comprehensive computational investigation on the impact of structural epigenetic features on CRISPR-Cas9 off-target activity. We use the Cas9 off-target activity database crisprSQL [39] and a comprehensive set of computational tools in this study. By doing so, we find that several nucleosome organization-related features attain higher correlation with off-target activity compared to the existing experimental epigenetic scores. In particular, this correlation is significantly higher for two Block Decomposition Method-based features [48, 49]. We also build physically inspired off-target activity prediction models that are purely based on empirical free energy estimates of the sgRNA-DNA heteroduplex and epigenetic features. This allows us to evaluate the impact of epigenetic features in the context of CRISPR-Cas9 activity model prediction. We find that said models take advantage of the computed nucleosome organization-related features but pay less attention to the commonly used experimental epigenetic scores.

## Results

### Spearman and Pearson correlation analysis

**Fig. 1:**
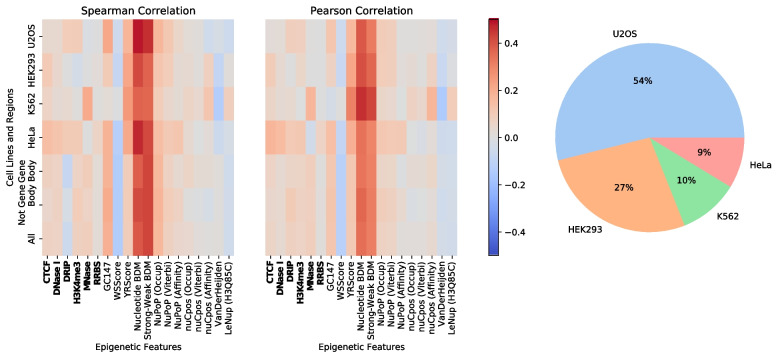
Heatmaps showing Spearman (left) and Pearson (middle) correlations between 19 epigenetic features and SpCas9 off-target cleavage activities. Red and blue colors represent positive and negative correlations, respectively. The 19 epigenetic features consists of six experimental epigenetic features (bolded) and 13 nucleosome organization-related scores. The first four rows in the heatmaps display cell line-specific correlations. The fifth and sixth row display correlations for off-target sites in gene body and non-gene body regions. The final row displays the overall correlation for the epigenetic features. (Right) Pie plot showing the dataset’s cell line composition including all cell lines that contribute more than $1\%$ to the crisprSQL dataset

Figure 1 shows two heatmaps denoting the Spearman and Pearson correlations of off-target cleavage activity with 19 epigenetic features (see exact values in Supplementary Table 1). The 19 epigenetic features consist of 6 experimental epigenetic features (names bolded in the figure) and 13 computed nucleosome organization-related features. Heatmap correlations are calculated for target sites in human cell lines HeLa, K562, HEK293 and U2OS from the CRISPR-Cas9 activity cleavage crisprSQL database [39]. To investigate whether correlation values vary between cell lines and genomic regions, heatmap correlations are displayed for all data, individual cell lines and gene/non-gene body regions. The rightmost pie chart shows the cell line composition of the dataset used for analysis. Overall, Spearman and Pearson correlations for the 19 epigenetic features considered range between -0.5 and 0.5. Only Nucleotide BDM and Strong-Weak BDM, i.e. BDM-based scores, exhibit highly positive correlations when considering all cell lines. Specifically, Nucleotide BDM has Spearman and Pearson correlations of 0.388 and 0.345, and Strong-Weak BDM has correlations of 0.423 and 0.310. Similar values are obtained for Nucleotide BDM and Strong-Weak BDM when considering cell lines individually. When filtering off-target sites by gene body and non-gene body regions, similar Spearman and Pearson correlations are observed across all epigenetic features. This indicates that correlations are not dependent on whether off-targets are in gene bodies. A similar trend is observed when considering each cell line separately (see Supplementary Figs. 2-4).

Table 1 highlights the correlation coefficients for the experimental epigenetic features shown in Fig. 1. In the table, Spearman/Pearson correlations between the six experimental features and off-target cleavage activities in any human cell lines range between -0.1 and 0.1. MNase, which is indicative of nucleosome occupancy rather than nucleosome positioning, has a Spearman and Pearson correlation of 0.08 and 0.08, respectively. Similar values are obtained for the various MNase-seq data across HeLa, K562 and U2OS (see Supplementary Figs. 2, 3 and 4, respectively).

**Table 1:** Spearman and Pearson correlation values between SpCas9 off-target cleavage activities and each experimental epigenetic scores for the crisprSQL dataset used in Fig. 1. The experimental epigenetic scores are CTCF, DNase I, DRIP, H3K4me3, MNase and RRBS

Experimental Epigenetic Feature	Spearman	Pearson
CTCF	0.07	0.06
DNase I	0.07	0.03
DRIP	-0.06	0.08
H3K4me3	0.07	0.07
MNase	0.08	0.08
RRBS	0.02	0.01

**Fig. 2:**
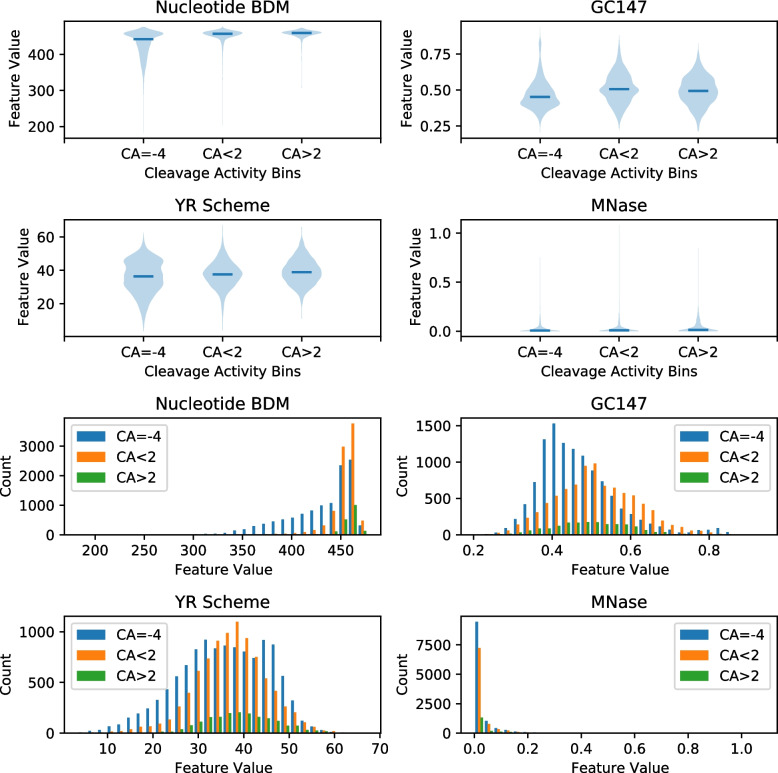
Violin (top) and distribution (bottom) plots for the epigenetic features with high Pearson correlation, namely Nucleotide BDM, GC147, YR Scheme and MNase. Cleavage activities (CA) are separated into three bins representing low ($\text {CA}=-4$, colored blue), medium ($\text {CA} \le 2$, colored orange) and high ($\text {CA} > 2$, colored green) cleavage activity. See violin and distribution plots for other epigenetic features in Supplementary Figs. 5 and 6, respectively

Figure 2 shows the violin and distribution plots for Nucleotide BDM, GC147, YR Scheme and MNase when splitting cleavage activities (CA) into three bins. These bins are $\text {CA}=-4$, $\text {CA} \le 2$ and $\text {CA} > 2$ (see Supplementary Figs. 5 and 6 for all epigenetic features). In the leftmost column for Nucleotide BDM, most off-target sites with low Nucleotide BDM value fall under the lowest cleavage activity bin CA=$-4$. The lowest cleavage activity datapoints are almost exclusively composed of augmented datapoints with sequence alignment-derived putative off-target sites. Such putative off-target sites are assigned the lowest cleavage activity value $\text {CA}=-4$ on the assumption that such sites have no off-target activity. Therefore, these datapoints do not carry experimentally derived cleavage activity labels. In addition, these datapoints constitute the larger fraction (52%) of all datapoints. A similar phenomenon is observed for Strong-Weak BDM (see Supplementary Figs. 5 and 6).

### Machine/Deep learning-based SHAP analysis

We saw that some computed nucleosome organization-related features correlate with CRISPR-Cas9 off-target activity. As a result, we sought to determine whether the aforementioned features also show patterns in machine and deep learning off-target cleavage activity prediction models. We also sought to investigate the importance of said features without the influence of explicitly encoded base pair identities. To achieve this, we built two models. The first model is an extreme gradient boosted (XGBoost) tree model. The second model is a convolutional neural network (CNN) model (see Supplementary Fig. 1 for neural network architecture). Both models take all 19 epigenetic features and three binding energy scores as input and predict off-target cleavage activities. Included in crisprSQL, the three energy scores represent free energy estimates used for estimating the DNA-RNA heteroduplex formation’s free energy. These energy terms have been generated by using the CRISPRspec [50] biophysical interaction model, which provides various binding energies scores (called CRISPRspec binding energy scores). These binding energies scores are further explained in the Methods section (see CRISPRspec section). The XGBoost and CNN models expect nucleosome organization-related features (scores) at base-pair resolution (23 scores per target site). sgRNA-DNA sequences were not included as input to both models. This is to avoid the interference of sequence features with epigenetic features when computing feature importance scores after training. Instead, we included the sgRNA-DNA sequences-derived CRISPRspec binding energy scores. When testing on the held out 20%, the XGBoost model achieves a Spearman and Pearson correlation of 0.419 and 0.617, respectively. The CNN model yields similar correlations, namely a Spearman and Pearson correlation of 0.424 and 0.594, respectively.

**Fig. 3:**
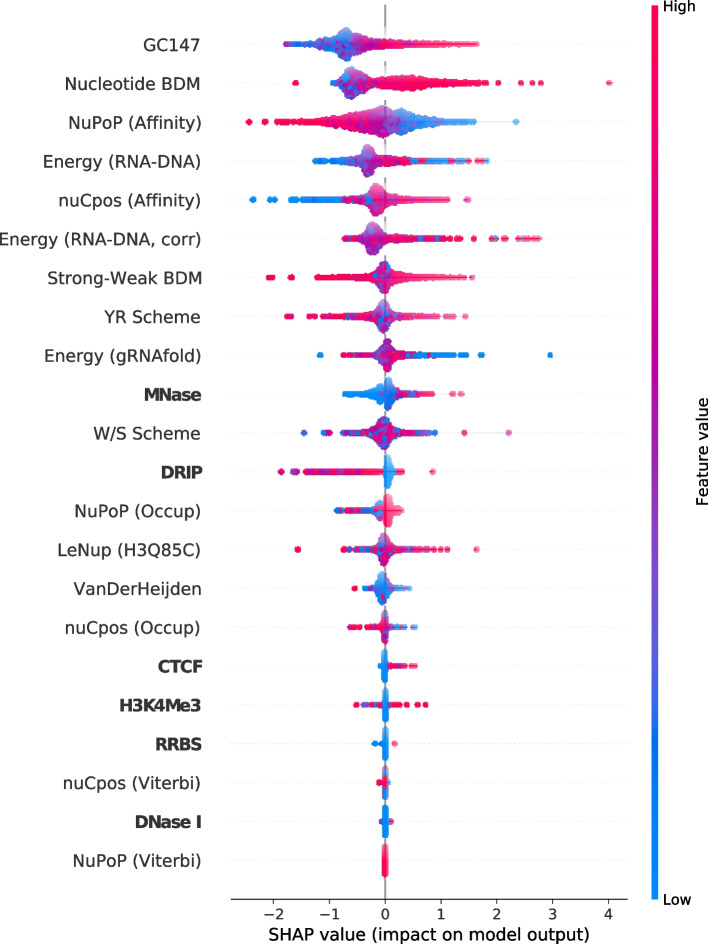
SHAP summary plot for the trained extreme gradient boosted (XGBoost) tree model. The model’s input consists of three CRISPRspec-derived energy terms, six experimental epigenetic scores (bolded), and 13 computed nucleosome organization-related scores. The three CRISPRspec-derived energy terms are $E_{\text {RNA-DNA}}$, $E_{\text {RNA-DNA}}^{\text {corr}}$ and *E*_gRNAfold_. The six experimental epigenetic scores are CTCF, DNase I, DRIP, H3K4me3, MNase and RRBS. The 13 computed nucleosome organization-related scores are GC147 [51], W/S scheme, YR scheme [52, 53], Strong-Weak BDM, Nucleotide BDM [48, 49], NuPoP (Occupancy), NuPoP (Affinity), NuPoP (Viterbi) [54], nuCpos (Occupancy), nuCpos (Affinity), nuCpos (Viterbi) [55], VanDerHeijden [56] and LeNup (H3Q85C) [57]. The base pair-resolved SHAP contributions for each data point are summed for each computed nucleosome organization-related score

**Fig. 4:**
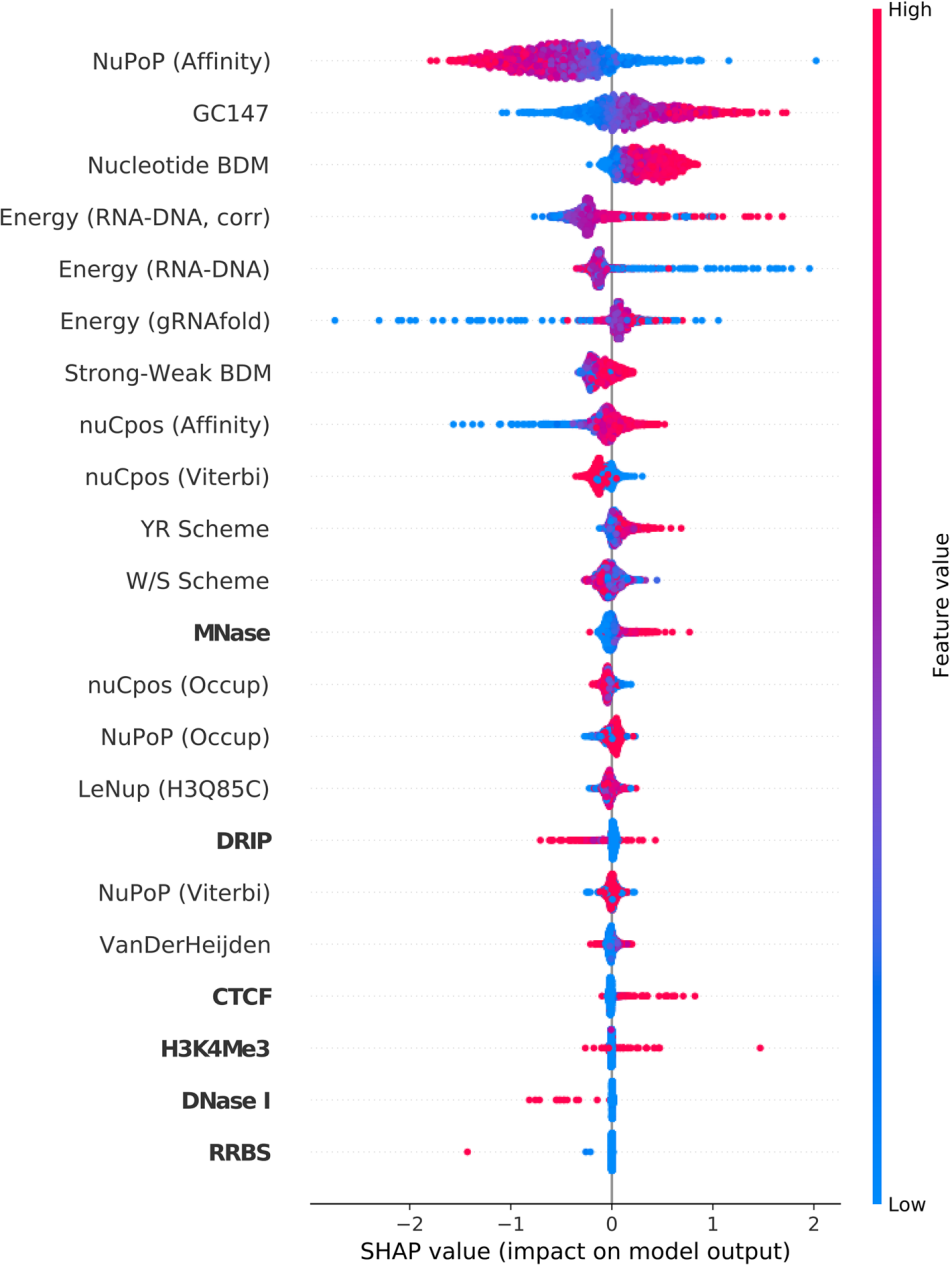
SHAP summary plot for the trained convolutional neural network (CNN) model (see Supplementary Fig. 1 for architecture details). The model’s input consists of three CRISPRspec-derived energy terms, six experimental epigenetic scores (bolded), and 13 computed nucleosome organization-related scores. The three CRISPRspec-derived energy terms are $E_{\text {RNA-DNA}}$, $E_{\text {RNA-DNA}}^{\text {corr}}$ and *E*_gRNAfold_. The six experimental epigenetic scores are CTCF, DNase I, DRIP, H3K4me3, MNase and RRBS. The 13 computed nucleosome organization-related scores are GC147 [51], W/S scheme, YR scheme [52, 53], Strong-Weak BDM, Nucleotide BDM [48, 49], NuPoP (Occupancy), NuPoP (Affinity), NuPoP (Viterbi) [54], nuCpos (Occupancy), nuCpos (Affinity), nuCpos (Viterbi) [55], VanDerHeijden [56] and LeNup (H3Q85C) [57]. The base pair-resolved SHAP contributions for each data point are summed for each computed nucleosome organization-related score

Next, we interpret the two model using SHAP (see Methods section) after training and evaluating the contributions of each input feature. To evaluate a model, a randomly drawn test dataset containing 2000 points is used. Figure 3 and Supplementary Fig. 7 show the resulting feature-based SHAP summary plot and base pair resolution heatmap, respectively, for the trained XGBoost model. An analogous summary plot and heatmap for the CNN model can be found in Fig. 4 and Supplementary Fig. 8. In the two SHAP summary plots, the distribution of SHAP value contributions is shown for every input feature present in the model. Model input features are ordered in decreasing SHAP feature importance. In other words, features at the top carry high SHAP feature importance, and features at the bottom carry low SHAP feature importance. In both SHAP summary plots, the SHAP feature importance of the six experimental epigenetic scores are not comparable to the nucleosome organization-related scores. In addition, the top five scores with highest SHAP feature importance include Nucleotide BDM and NuPoP (Affinity). These two features display similar correlations between feature value and SHAP value across Figs. 3 and 4. Notably, low Nucleotide BDM values and high NuPoP (Affinity) values correspond to negative impact on off-target activity. As for the three CRISPRspec binding energy scores, they attain comparable SHAP feature importance to top-performing nucleosome organization-related scores in both models.

## Discussion

MNase-seq, a common genome-wide experimental technique, appear to be an attractive option for obtaining raw nucleosome occupancy data. In addition, nucleosome occupancy data may be indicative of CRISPR-Cas9 off-target activity. On account of this, we sought to obtain MNase-seq data from NucPosDB [58] where available for human cell lines. Nonetheless, we found MNase-seq data only for U2OS, K562 and HeLa in NucPosDB. In particular, MNase-seq data must be measured for each cell line of interest in order to curate sufficient data for analysis. This makes MNase-seq data cell-based and difficult to obtain. Such qualities are the opposite of computed nucleosome organization-related scores, which are not only genome-wide but also easy to obtain and cell-line independent.

The experimental features CTCF, DNase I, RRBS and H3K4me3 are commonly used as input features in multiple state-of-the-art deep learning-based CRISPR-Cas9 off-target activity prediction tools [33, 35, 59]. Despite this, we can see in Fig. 1 and Table 1 that BDM-based scores attain much higher Spearman and Pearson correlations with off-target cleavage activities. This is in contrast to the six experimental epigenetic features listed in Table 1, which do not strongly correlate with cleavage activity.

Scrutinizing the distributions in Fig. 2, we observe that most off-target sites with low Nucleotide BDM value fall under the lowest cleavage activity bin CA=$-4$. Moreover, crisprSQL is augmented with sequence alignment-derived putative off-target sites. Such putative sites are assigned the lowest cleavage activity value CA$=-4$ on the assumption that such sites have no off-target activity. As a result, among the putative sites, Nucleotide BDM is better at separating sites without activity from sites with activity, compared to other epigenetic features. The aforementioned observations can be explained by the correspondence between low Nucleotide BDM values and proximity to nucleosome dyad positions [49]. Since these positions are blocked by nucleosomes, they are inaccessible for Cas9 binding and cleavage, thus resulting in low off-target activity. This is a possible explanation on why these off-target sites have not been experimentally identified as active. In practice, the application of Nucleotide BDM for data filtering can be useful when preparing data for CRISPR-Cas9 off-target model training. This is because such a filtering might help resolve any class imbalances between experimentally measured and putative off-targets. Moreover, Nucleotide BDM is a fundamental property of the 147bp nucleosomal DNA context which is not dependent on any training dataset. Deeper understanding of why augmented datapoints (i.e., lowest cleavage activity datapoints) have no off-target activity is currently lacking. To the best of our knowledge, there has not been any existing target sequence-based measure that could separate augmented datapoints from experimentally-derived datapoints. Figure 2 indicates that low values of Nucleotide BDM can separate these augmented datapoints remarkably well compared to other similar measures.

In Figs. 3 and 4, the six experimental scores’ low SHAP feature importance demonstrates that they are inappropriate for informing off-target cleavage activity prediction models. This corroborates with the Spearman and Pearson correlation values in Table 1. The top five scores with highest SHAP feature importance include Nucleotide BDM and NuPoP (Affinity). The two features show similar correlations between feature value and SHAP value across the two plots. Notably, low Nucleotide BDM values and high NuPoP (Affinity) values correspond to negative impact on off-target activity. This observation corroborates the fact that such feature values often are signals of positioned nucleosomes. It follows that information in BDM-based scores and NuPoP (Affinity), alongside other nucleosome organization-related scores, are well suited for informing off-target cleavage activity prediction models. GC147’s importance as a feature in both machine learning models is in agreement with latest findings [60] that CRISPR-Cas9 bends DNA to read its sequence. Specifically, DNA bendability is very highly correlated with GC content [61]. Such a fact could explain the findings of the SHAP summary plot, namely that high GC147 has a positive impact on (off-)target cleavage activity. The three CRISPRspec binding energy scores contribute significantly towards model predictions in both models, which confirms these scores’ usefulness for CRISPR-Cas9 off-target activity prediction. Despite interesting structures in the heatmaps of Supplementary Figs. 7 and 8, a thorough analysis of such structures is beyond the scope of this study. In off-target prediction, the most suitable use case for Nucleotide BDM and other relevant measures is to incorporate them in ‘complete’ deep learning models. Together with measures like NuPoP (Affinity) and GC147, they can be combined with the guide-RNA-(off-)target DNA sequence pair as input to such models.

Interestingly, only BDM-based scores have noticeable correlation with (off-)target activities. However, the NuPoP (Affinity) score has comparable SHAP feature importance to Nucleotide BDM score in both machine learning models considered in this work. Supplementary Fig. 9 shows that the correlation between NuPoP (Affinity) and Nucleotide BDM is relatively low. This observation agrees with the finding that only one of the two scores (Nucleotide BDM) correlates with (off-)target activity. However, it does not alone explain why the other score, NuPoP (Affinity), is still a comparably impactful feature in both machine learning models. To investigate this further, we obtained SHAP dependence plots for both models, which include NuPoP (Affinity) and Nucleotide BDM (see Supplementary Figs. 11 and 12). These plots show that a given NuPoP (Affinity) value can have different impact (importance) based on the corresponding Nucleotide BDM value of a data point. This last observation explains why NuPoP (Affinity) does not noticeably correlate with (off-)target activity, yet is an important feature for both models, since they include NuPoP (Affinity) and Nucleotide BDM scores simultaneously.

Our results indicate that only a few out of 13 nucleosome organization-related scores show noticeable correlation with (off-)target activity or are important for model predictions. Most of these high-importance features ‘measure’ nucleosome affinity rather than nucleosome occupancy. Consequently, we speculate that the influence of high nucleosome affinity on Cas9 (off-)target activity exceeds that of high nucleosome occupancy. Such speculation is in concordance with the low impact of the NuPoP (Occupancy) score (see Figs. 3 and 4) on model predictions.

## Conclusions

For all off-target sites featured in the crisprSQL Cas9 off-target database, we obtained 19 epigenetic features, 15 of which were newly considered. The introduced computed features characterize nucleosome organization, and include features based on BDM-based or NuPoP (Affinity). We also considered six experimental epigenetic features, namely CTCF, DNase I, DRIP, H3K4me3, MNase and RRBS. We showed that the computed features exhibited considerably larger correlation with off-target cleavage activity when compared to the six experimental epigenetic features. Interestingly, only the features CTCF, DNase I, H3K4me3 and RRBS have been frequently used in deep learning-based off-target activity prediction models. As expected, nucleosome positioning negatively impacts off-target activity. This is shown by the low Nucleotide BDM scores assigned to putative off-target sites with no detectable off-target activity. We explain this phenomenon by the presence of positioned nucleosomes which inhibit Cas9 binding. Including empirical estimates of sgRNA-DNA heteroduplex binding energies as inputs, we constructed an XGBoost tree and a CNN model. The two models were used in order to gain feature importance values of all epigenetic features. Next, we created a SHAP summary plot for each model, with feature contribution quantified by the average SHAP feature importance value across data points. The plots showed GC147, Nucleotide BDM and NuPoP (Affinity) as features among the top five which contribute most to the model’s output in both models. Their importance in the two models are unlike the six experimental epigenetic scores. We uploaded the off-target cleavage activity dataset used in order to make the experimental epigenetic and computed nucleosome organization-related scores available for further research. This dataset can be found as a compressed Parquet file at https://crisprsql.com/downloads/260520_putative_nucleosomal.parquet.gz. For future work, computed scores could be combined with target sequence and binding energy features in more robust and complete CRISPR-Cas9 off-target activity prediction models. Notably, BDM-derived and NuPoP scores could be used in such models. It would also be fruitful to scrutinize whether BDM-derived and NuPoP (Affinity) are also predictive of off-target activity in other CRISPR-Cas systems.

## Methods

### crisprSQL

The crisprSQL database consists of experimental off-target sites and cleavage activities from 15 human CRISPR-Cas9 off-target studies. In order to conduct a comprehensive investigation on the effect of epigenetics and nucleosomes on CRISPR-Cas9 off-target activity, we utilize crisprSQL [39]. crisprSQL is an up-to-date Cas9 off-target database containing sequence and epigenetic information for over 25,000 gRNA-off-target pairs from various human and rodent cell lines. Different experimental techniques were used to measure off-target activity in different studies. Consequently, we combine the experimental off-target cleavage activities from each study by applying a Box-Cox transformation. The transformation is such that the resulting combined cleavage activity data approximates a Gaussian with $\text {mean}=0$ and $\text {standard deviation}=2$, as suggested in [39]. Transformed values were clipped to the $[-4, 4]$ range, with cleavage activity values below the lowest reported assay accuracy of $10^{-5}$ set to $-4$. We furthermore augment the sites in crisprSQL with those in the respective genome which have less than seven mismatches compared to any experimental data point. These data points are assumed to have no off-target activity ($\text {CA}=-4$). Using the sequence alignment tool batmis for this [62], we generate 226,682 augmented data points. This results in a total of 251,854 data points in our dataset. In summary, the above steps yield a crisprSQL-derived dataset which was augmented with putative off-targets.

### Experimental nucleosome occupancy data

The NucPosDB database [58] consists of experimental nucleosome positioning and occupancy data aggregated from various biological publications. Micrococcal Nuclease digestion with deep sequencing (MNase-seq) data are indicative of nucleosome occupancy and chromatin accessibility. In addition, MNase-seq may be indicative of CRISPR-Cas9 off-target activity. Consequently, MNase-seq data for human cell lines present in crisprSQL are extracted from NucPosDB where available. This yields three HeLa (GSM1602359 [63], GSM2680344-2680347 [64]), five K562 (GSE78984 [65], GSM920557 [66], GSM2083137-2083140 [65]) and two U2OS (GSM1838910-1838911 [67]) MNase-seq tracks. Such tracks for HeLa, K562 and U2OS are then used for annotating crisprSQL off-target sites observed in the corresponding cell line.

### Adding epigenetic scores

To construct the dataset for our study, we extract the 23bp target DNA sequence and 169bp target-centered sequence context for all gRNA-target pairs in crisprSQL. We also extract the experimental epigenetic (i.e., CTCF, DNase I, H3K4me3 and RRBS) scores and the normalized off-target cleavage activity for all aforementioned gRNA-target pairs. To create a single experimental epigenetic MNase feature from the cell-specific tracks, we first average HeLa data from replicate tracks GSM2680344 and GSM2680345. Next, we average U2OS data from replicate tracks GSM1838910 and GSM1838911, and directly adopt GSM2083140 for K562. We then linearly rescale each of the three resulting sets of MNase data to [0, 1], and concatenate the sets together into a single feature. We assign zeros to off-target sites with no available MNase data. In summary, this yields a crisprSQL-derived dataset with 6 experimental epigenetic scores for each of the experimental and putative off-target sites.

### Adding nucleosome organization-related scores

Various existing procedural and training-based data-driven computational tools are used for predicting nucleosome organization-related scores such as nucleosome occupancy and positioning. Whereas training-free procedural tools are adopted wherever available, only three recently developed training model-based tools, namely, NuPoP [54], nuCpos [55] and LeNup, were adopted. This is because these tools attain similar performances to the gold standard nucleosome occupancy model from Kaplan et al. [68, 69]. Alternatively, they use chemical cleavage-based nucleosome positioning data [55, 70] which have higher resolution compared to the MNase-seq data used in the gold standard model.

We further augment the crisprSQL dataset with nucleosome organization-related scores. This is done by computing nucleosome occupancy and/or positioning-related scores for each base pair in the 23bp target sequence for all off-target sites. To compute a variety of scores for each 169bp sequence context, we use a comprehensive set of nucleosome organization-related tools. The names of these tools are GC content (abbreviated GC147) [51], W/S scheme [52, 53], YR scheme [52, 53], Van Der Heijden [56], Block Decomposition Method (BDM) [48, 49], NuPoP [54], nuCpos [55], and LeNup [57]. Note that nucleosome organization-related tools like BDM [48] cannot handle ‘N’-containing input sequences. As a result, the dataset used in this study only consider off-target sites with non-‘N’-containing sequence contexts.

The following subsections details how each tool is used for computing one or more nucleosome organization-related scores. Since NuPoP and nuCpos both output histone affinity, nucleosome occupancy, and Viterbi scores, we include all three scores as separate features for both tools. We also derive Nucleotide BDM and Strong-Weak BDM scores from BDM. As a result, the 8 tools above generate 13 computed scores. In summary, the above steps yield a crisprSQL-derived dataset which was augmented with putative off-targets. In terms of features, it has a total of 6 experimental epigenetic and 13 nucleosome organization-related computed features. We further refer to these 19 features as epigenetic features.

#### GC content

GC content (or GC147 as abbreviated here for clarity) is a simple training-free measure. It is defined as the fraction of guanine and cytosine residues present in the 147bp nucleosomal sequence around a given nucleotide. Details on the use of GC content for predicting nucleosome occupancy can be found in the supplementary material.

We compute base pair-resolved GC147 values for each (off-)target site in the crisprSQL dataset. To do this, we slide a 147bp window across the (off-)target site’s 169bp context sequence, thereby obtaining 23 subsequences of length 147. A GC147 value is then computed for each of these subsequences.

#### W/S and YR schemes

W/S and YR schemes are training-free scores used for the prediction of rotational and translational nucleosome positioning, respectively [52]. The two schemes are available on the web platform nuMap [53], and are based on sequence-dependent DNA anisotropy. Details regarding how W/S and YR schemes work can be found in the supplementary material.

We compute base pair-resolved W/S and YR Scheme values for each (off-)target site in the crisprSQL dataset. The general approach for doing this is identical to that of GC147. Namely, we slide a 147bp window across the (off-)target site’s 169bp context sequence, thereby obtaining 23 subsequences of length 147. The only difference is that we use W/S and YR Scheme instead of GC147 when computing values for each of the 23 subsequences.

#### Van Der Heijden algorithm

In reference [56], the authors propose a method for predicting the intrinsic nucleosome position of a genome based on statistical mechanics. We abbreviate this method as VanDerHeijden. Details regarding how VanDerHeijden works can be found in the supplementary material.

We compute base pair-resolved VanDerHeijden values for all (off-)target sites in the crisprSQL dataset. To compute a VanDerHeijden score for a given (off-)target site, we first obtain the 169bp context sequence of the given site. The context sequence is then padded with 73 A nucleotides on both ends, and then passed into the Van Der Heijden algorithm (see Supplementary Material). Reading the middle 23 values in the array of 169 values produced by the algorithm then yields the base pair-resolved values. We use the following hyperparameters for VanDerHeijden:

- a nucleosome positioning window of $N=147$,
- probability amplitude $$B=0.16$$,
- dinucleotide periodicity $p=10.1$, and
- chemical potential $$\mu = -0.6$$.

An implementation of the algorithm can be found at https://github.com/JvN2/NucTool.

#### Block decomposition method-based measures

Many recent nucleosome occupancy tools such as NuPoP are statistical and entropy-based. However, such tools often require the use of experimental nucleosome occupancy data for the training of many parameters in the model, which is computationally expensive. To resolve this, we can use the Block Decomposition Method (BDM) [48], which is a training-free method for approximating the algorithmic complexity of sequences. A consequence of this definition is that repetitive sequences, e.g., “ATATATATAT”, have low BDM values. A recent study [49] showed that BDM scores of 147bp candidate DNA sequences carry valuable information related to nucleosome organization.

Based on BDM, we derive Nucleotide BDM, which computes the BDM of the 147bp DNA string. We also derive Strong-Weak BDM, which applies the strong-weak transformation before computing the BDM of the resulting modified string. The strong-weak transformation replaces ‘G’ and ‘C’ with ‘S’ (Strong) and ‘A’ and ‘T’ with ‘W’ (Weak) in the DNA string. We compute base pair-resolved Nucleotide BDM and Strong-Weak BDM values for each (off-)target site in the crisprSQL dataset. The general approach for doing this is identical to that of GC147. Namely, we slide a 147bp window across the (off-)target site’s 169bp context sequence, thus obtaining 23 subsequences of length 147. We then use PyBDM, a Python [71] implementation of BDM, to compute Nucleotide BDM and Strong-Weak BDM values for each of the 147bp subsequences. The Python implementation of BDM can be found in https://github.com/sztal/pybdm.

#### NuPoP

Using a duration Hidden Markov Model (dHMM), NuPoP [54] predicts nucleosome positioning and occupancy. NuPoP accounts for the different linker length distributions or base compositions in different eukaryotes in order to make better predictions [72]. Details on NuPoP can be found in the supplementary material. An implementation of NuPoP can be found at https://github.com/jipingw/NuPoP.

We compute base pair-resolved NuPoP (Affinity), NuPoP (Occupancy) and NuPoP (Viterbi) values for each (off-)target site in the crisprSQL dataset. First, the 294,989 context sequences in the crisprSQL dataset were split into 9 sets of size 31,645 and 1 set of 10,184. This is to accommodate the fact that NuPoP requires an input sequence length of at least 1000bp. Long strings of length $147 + (147 + 169) * 31,645 = 9,999,967$ were created for the first 9 set by adding 147 A nucleotides between each context sequence. To remove end effects, the long string also contains 147 A nucleotides both before the first context sequence and after the last context sequence. In the same way, a short string of length $147 + (147 + 169) * 10,184 = 3,218,291$ is created for the final set. The 10 long strings are then fed into the NuPoP R package individually using the predNuPoP function. This gives rise to 10 TSV files containing the base pair-resolved histone binding affinity, occupancy and Viterbi values. When calling predNuPoP, we use parameters species=1 and model=4.

#### nuCpos

Building on NuPoP, nuCpos [55] is a recent dHMM-based algorithm for predicting nucleosome positioning. nuCpos uses the same training and inference algorithms as NuPoP. However, it improves upon NuPoP by using high-resolution H3Q85C-seq budding yeast data [70] instead of the low-resolution MNase-seq data. Similar to NuPoP, nuCpos produces histone binding affinity, predicted nucleosome occupancy and Viterbi scores. More details on the algorithm can be found in [55]. An implementation of nuCpos can be found at https://github.com/hkatomed/nuCpos.

We compute base pair-resolved nuCpos (Affinity), nuCpos (Occupancy) and nuCpos (Viterbi) values for each (off-)target site in the crisprSQL dataset. The nuCpos R package has similar input-output interfaces to NuPoP. Consequently, we use the same approach as that described for NuPoP above in order to produce these base pair-resolved values. When calling predNuCpos, we use parameters species="c", smoothHBA=FALSE and ActLikePredNuPoP=TRUE.

#### LeNup

In light of the recent rise of state-of-the-art deep learning methods for data-based models, LeNup uses a convolutional neural network (CNN) with gated Inception-like modules [73, 74]. LeNup is used for nucleosome positioning prediction in a variety of eukaryotic genomes [57]. The original implementation of LeNup is available at https://github.com/biomedBit/LeNup.

LeNup was originally trained for separating nucleosomal and non-nucleosomal DNA. Consequently, we retrained the neural network used in LeNup using high resolution H3Q85C chemical cleavage-seq [70] yeast data. Because of this modification, we will refer to this measure as LeNup (H3Q85C). The retrained PyTorch [75] model can be found at https://github.com/jeffmak/crispr-cas9-epigenetics. We compute base pair-resolved LeNup (H3Q85C) values for all (off-)target site in the crisprSQL dataset. For each (off-)target site, we one-hot encode its context sequence and pass it into the PyTorch model, which outputs the base pair-resolved value.

### Correlation and distribution analysis

We compute the Spearman and Pearson correlations with off-target cleavage activities for all epigenetic features. This enables us to examine the relationship between each epigenetic feature and off-target cleavage activity, and to identify features which significantly correlate with off-target activity. We also consider whether such correlations vary between gene and non-gene bodies or across cell lines. The calculation of gene bodies is not cell line dependent. The nucleosome organization-related scores are at base-pair resolution. Consequently, we take the mean of the values at each (off-)target if the score is not binary and the median of the values otherwise. Using the dataset which was augmented with putative off-targets, we separate the data points into lowest ($\text {CA}=-4$), low ($\text {CA} \le 2$) and high ($\text {CA}>2$) cleavage activity. We also visualize the epigenetic score distributions for these data points. In order to compare cleavage frequencies across studies, we use the nonlinear Box-Cox transformation [76] to transform cleavage rates. We transform cleavage rates to approximate a Gaussian with zero mean and standard deviation $\sigma = 2$ for each study individually. To achieve a fixed value range and treat outliers efficiently, this distribution has been clipped at $-2\sigma$ and $2\sigma$. This has been used in the literature [77, 78] before. Based on these, we separate the data points into lowest cleavage activity ($\text {CA}=-2\sigma =-4$), low cleavage activity ($\text {CA} \le \sigma = 2$) and high cleavage activity ($\text {CA}>\sigma$).

### CRISPRspec

The crisprSQL database includes estimates for the free energy of the DNA-RNA heteroduplex generated by the CRISPRspec [50] biophysical interaction model. These interaction energies are features derived from secondary structures. These features shape the thermodynamic advantage to gRNA-DNA hybrid formation upon binding of the gRNA-Cas9 complex to the off-target site. Computationally, for a given (off-)target region, CRISPRspec uses four empirical free energy contributions terms, namely:

- a PAM-dependent correcting factor $\delta _{\text {PAM}}$,
- free energy $\Delta G_H^{\text {RNA:DNA}}$ from hybridizing the gRNA and target strand, weighted by a position-wise estimate of the Cas9 influence in the binding,
- free energy $\Delta G_U^{\text {RNA:RNA}}$ from forming the secondary structure of the 20nt gRNA spacer sequence, computed using RNAFold,
- free energy $\Delta G_O^{\text {DNA:DNA}}$ from forming the dsDNA duplex from the target and non-target DNA strands.

These four terms are used for computing the total binding free energy

$$\begin{aligned} \Delta G_B = \delta _{\text {PAM}}(\Delta G_H^{\text {RNA:DNA}} - \Delta G_U^{\text {RNA:RNA}} - \Delta G_O^{\text {DNA:DNA}}). \end{aligned}$$

From the values given in the crisprSQL database, we calculate three key energy features to be included in our model, namely

- $$E_{\text {RNA-DNA}} = \delta _{\text {PAM}} \Delta G_H^{\text {RNA:DNA}},$$
- $$E_{\text {RNA-DNA}}^{\text {corr}} = \delta _{\text {PAM}}(\Delta G_H^{\text {RNA:DNA}} - \Delta G_O^{\text {DNA:DNA}}),$$
- $$E_{\text {gRNAfold}} = \Delta G_U^{\text {RNA:RNA}}.$$

### Model and SHAP

CRISPR recently saw an increase in computational tools for Cas9 off-target activity prediction [79], with recent tools using machine and deep learning techniques [33, 35, 59, 80, 81]. To determine how all 19 epigenetic scores relate to off-target activity within a Cas9 off-target cleavage activity prediction model, we build two machine learning models. The first one is an extreme gradient boosted (XGBoost) tree model [82], and the second one a convolutional neural network (CNN) model. These models take three CRISPRspec-derived energy features [50], experimental epigenetic features and nucleosomal organization-related features. The CNN’s model architecture is similar to DeepCRISPR’s [33] Siamese neural network, but lacks the sequence arm (see Supplementary Fig. 1 for details on the architecture). Any nucleosome organization-related feature is calculated at base pair resolution leading to 23 values for an (off-)target DNA. In contrast, the mean value across the 23 (off-)target base pairs is presented for any experimental epigenetic feature.

Regarding training and evaluation for the XGBoost and CNN models, the dataset is randomly split into a training dataset and test dataset. A ratio of 80%-20% is used for the splitting. The train-test split is done in a way so as to ensure equal amounts of experimentally measured and augmented data in both datasets. For XGBoost, the tree model is trained for 70 epochs, where a new training batch with 50,000 data points is sampled in each epoch. We chose hyperparameters eta=0.5, colsample_bytree=0.7, max_depth=7. As for CNN, the model is trained for 70 epochs, where a new training batch with 35,000 data points is sampled in each epoch. We use hyperparameters lr=0.001, batchnorm_momentum=0.1, together with early stopping. For both models, bootstrap sampling ensures that each training batch contains equal amounts of active ($\text {CA}>-4$) and inactive/putative ($\text {CA}=-4$) (off-)targets. We then use the Shapley Additive Explanation (SHAP) library’s Tree Explainer and Deep Explainer [83]. We use these explainers on a batch of 10,000 datapoints randomly sampled from the test data. This allows us to measure the contribution of each input feature towards the XGBoost and CNN model’s prediction respectively. Contributions for each input features are then visualized using SHAP summary plots. When creating the SHAP summary plots, for each data point, we compute the SHAP contribution of each computed feature in the SHAP summary plots. The SHAP contribution for each computed feature is computed by summing up the corresponding base pair-resolved SHAP contributions.

## Supplementary Information

**Additional file 1: Supplementary Table 1** Spearman and Pearson correlation values between epigenetic features and SpCas9 off-target cleavage activities. **Supplementary Figure 1** Convolutional neural network architecture used for CRISPR-Cas9 off-target activity prediction. **Supplementary Figure 2** Heatmaps showing Spearman and Pearson correlations between epigenetic features and Cas9 off-target cleavage activities for HeLa cell line data. **Supplementary Figure 3** Heatmaps showing Spearman and Pearson correlations between epigenetic features and Cas9 off-target cleavage activities for K562 and U2OS cell line data. **Supplementary Figure 4** Heatmaps showing Spearman and Pearson correlations between epigenetic features and Cas9 off-target cleavage activities for K562 and U2OS cell line data. **Supplementary Figure 5** Violin plots for all epigenetic features. **Supplementary Figure 6** Distribution plots for all epigenetic features. **Supplementary Figure 7** Heatmap showing the mean absolute value of the SHAP values for the extreme gradient boosted tree's base pair-resolved input features. **Supplementary Figure 8** Heatmap showing the mean absolute value of the SHAP values for the convolutional neural network's base pair-resolved input features. **Supplementary Figure 9** Spearman and Pearson Correlations between NuPoP (Affinity) and Nucleotide BDM across different cell lines (U2OS, HEK293, K562, HeLa) and regions (Gene Body, Not Gene Body) for the dataset used in Fig. 1. **Supplementary Figure 10** Bar plot showing Spearman and Pearson correlations between 19 epigenetic features and SpCas9 on-target cleavage activities for all cell lines that contribute more than 1% to the crisprSQL dataset. **Supplementary Figure 11** SHAP dependency plots for GC147, Nucleotide BDM and NuPoP (Affinity) for XGBoost model. **Supplementary Figure 12** SHAP dependency plots for GC147, Nucleotide BDM and NuPoP (Affinity) for CNN model.

## Data Availability

The crisprSQL database is available at http://www.crisprsql.com, where the full data set can be downloaded in CSV format. The dataset used for analysis in this paper is available as a compressed Parquet file at https://crisprsql.com/downloads/260520_putative_nucleosomal.parquet.gz, where the full data set can be downloaded in parquet format. Users are not required to log in to access any of the database features. Sample Python scripts for using the XGBoost and CNN models are available at https://github.com/jeffmak/crispr-cas9-epigenetics.

## Abbreviations

CRISPR: Clustered regularly interspaced short palindromic repeats
gRNA: Guide RNA
XGBoost: Extreme Gradient Boost
CNN: Convolutional neural network
SHAP: Shapley Additive Explanation
