# Supplementary material for: Comprehensive computational analysis of epigenetic descriptors affecting CRISPR-Cas9 off-target activity
Source: BMC Genomics. 2022 Dec 6;23:805. doi: 10.1186/s12864-022-09012-7 (PMC9724382; doi:10.1186/s12864-022-09012-7)
Supplement: Supplementary file 1 — Additional file 1: Supplementary Table 1 Spearman and Pearson correlation values between epigenetic features and SpCas9 off-target cleavage activities. Supplementary Figure 1 Convolutional neural network architecture used for CRISPR-Cas9 off-target activity prediction. Supplementary Figure 2 Heatmaps showing Spearman and Pearson correlations between epigenetic features and Cas9 off-target cleavage activities for HeLa cell line data. Supplementary Figure 3 Heatmaps showing Spearman and Pearson correlations between epigenetic features and Cas9 off-target cleavage activities for K562 and U2OS cell line data. Supplementary Figure 4 Heatmaps showing Spearman and Pearson correlations between epigenetic features and Cas9 off-target cleavage activities for K562 and U2OS cell line data. Supplementary Figure 5 Violin plots for all epigenetic features. Supplementary Figure 6 Distribution plots for all epigenetic features. Supplementary Figure 7 Heatmap showing the mean absolute value of the SHAP values for the extreme gradient boosted tree's base pair-resolved input features. Supplementary Figure 8 Heatmap showing the mean absolute value of the SHAP values for the convolutional neural network's base pair-resolved input features. Supplementary Figure 9 Spearman and Pearson Correlations between NuPoP (Affinity) and Nucleotide BDM across different cell lines (U2OS, HEK293, K562, HeLa) and regions (Gene Body, Not Gene Body) for the dataset used in Fig. 1. Supplementary Figure 10 Bar plot showing Spearman and Pearson correlations between 19 epigenetic features and SpCas9 on-target cleavage activities for all cell lines that contribute more than 1% to the crisprSQL dataset. Supplementary Figure 11 SHAP dependency plots for GC147, Nucleotide BDM and NuPoP (Affinity) for XGBoost model. Supplementary Figure 12 SHAP dependency plots for GC147, Nucleotide BDM and NuPoP (Affinity) for CNN model. [file 12864_2022_9012_MOESM1_ESM.pdf]

Supplementary data for “Comprehensive computational analysis of epigenetic descriptors affecting CRISPR-Cas9 off-target activity”

Jeffrey Mak, Florian Störtz and Peter Minary

1 Supplementary Tables and Figures

Extending Figure 1, Supplementary Table 1 lists the off-target cleavage activity Spearman and Pearson correlation values for all experimental epigenetic and computed nucleosome organization-related features. Figure 1 shows the architecture of the convolutional neural network used for CRISPR-Cas9 off-target activity prediction and SHAP value analysis. Extending Figure 1, Supplementary Figures 2, 3 and 4 show the cell line-based heatmaps indicating Spearman and Pearson correlations between the epigenetic features and CRISPR-Cas9 off-target activity for cell lines HeLa, K562 and U2OS, respectively. Extending Figure 2, Supplementary Figures 5 and 6, respectively, show the violin and distribution plots for CRISPR-Cas9 off-target cleavage activity for all 19 experimental epigenetic features and computed nucleosome organization-related, with the experimental epigenetic features highlighted in bold. Extending Figures 3 and 4, Supplementary Figures 7 and 8 visualize the SHAP contribution of each input feature in a trained XGBoost and CNN model, respectively, both of which predict CRISPR-Cas9 off-target activity, where all computed nucleosome organization-related scores are base pair-resolved.

Using only the 'on-target' datapoints that correspond to guide-RNA–on-target DNA sequence pairs, Supplementary Figures 10 shows an overall correlation analysis. It can be seen that Nucleotide (and Strong-Weak) BDM still show the highest Spearman correlation with on-target cleavage activity, even though the difference in correlation values is not as pronounced as found for the off-target cleavage activity dataset.

**Epigenetic Feature**	**Spearman**	**Pearson**
Strong-Weak BDM	**0.423**	0.310
Nucleotide BDM	0.388	**0.345**
GC147	0.191	0.117
NuPoP (Occupancy)	0.167	0.068
YR Scheme	0.087	0.108
**MNase**	0.082	0.083
NuPoP (Viterbi)	0.078	0.060
**H3K4me3**	0.075	0.066
**CTCF**	0.070	0.059
**DNase I**	0.065	0.033
NuPoP (Affinity)	0.048	0.011
nuCpos (Occupancy)	0.040	0.015
**RRBS**	0.022	0.009
nuCpos (Viterbi)	0.014	0.015
VanDerHeijden	0.009	-0.036
nuCpos (Affinity)	0.005	0.037
LeNup (H3Q85C)	-0.050	-0.043
**DRIP**	-0.059	0.076
W/S Scheme	-0.141	-0.122

Supplementary Table 1: Spearman and Pearson correlation values between epigenetic features and SpCas9 off-target cleavage activities. Epigenetic features include all nucleosome organization-related scores and six experimental epigenetic scores (bolded). The correlations were derived using the said scores and cleavage activities for all datapoints defined in the Materials and Methods section. The features are sorted by decreasing Spearman correlations and the highest Spearman and Pearson correlation values are highlighted in bold.

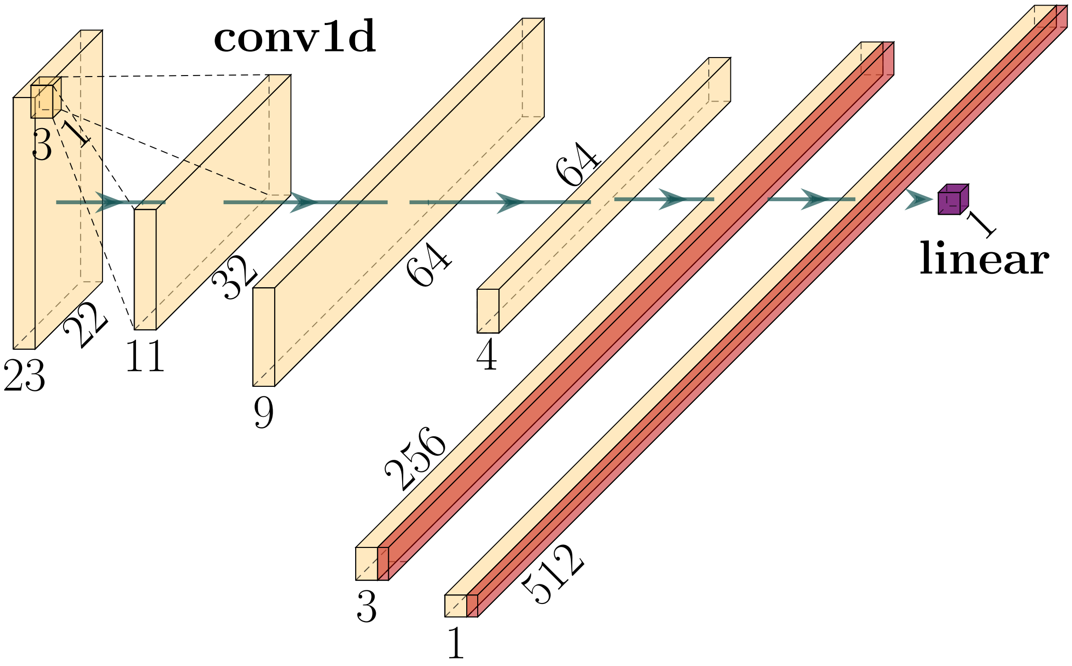

Supplementary Figure 1: Convolutional neural network architecture used for CRISPR-Cas9 off-target activity prediction as mentioned in the Methods section. The architecture is implemented in PyTorch [1]. The input to the neural network is a 23bp $\times$ 22 features input matrix, and the output is a scalar value indicative of the CRISPR-Cas9 off-target activity prediction. The architecture consists of five one-dimensional convolutional (Conv1D) layers followed by one fully connected layer. The first layer is a Conv1D layer with 32 channels, 3×3 kernel size, stride of 2 and padding of 0, followed by leaky rectified linear unit activation (LeakyReLU) [2] with a negative slope of 0.2. The second layer is a Conv1D layer with 64 channels, 3×3 kernel size, stride of 1 and padding of 0, followed by LeakyReLU [2] with a negative slope of 0.2. The third layer is a Conv1D layer with 128 channels, 3×3 kernel size, stride of 2 and padding of 0, followed by 1D batch normalization and subsequently LeakyReLU [2] with a negative slope of 0.2. The fourth layer is a Conv1D layer with 256 channels, 3×3 kernel size, stride of 1 and padding of 0, followed by 1D 3×3 max pooling with padding of 1 and stride of 1, and subsequently rectified linear unit activation (ReLU). The fifth layer is a Conv1D layer with 512 channels, 2×2 kernel size, stride of 1 and padding of 0, followed by 1D 3×3 max pooling with padding of 1 and stride of 1, and subsequently rectified linear unit activation (ReLU). The final layer is a fully connected layer which outputs a scalar value.

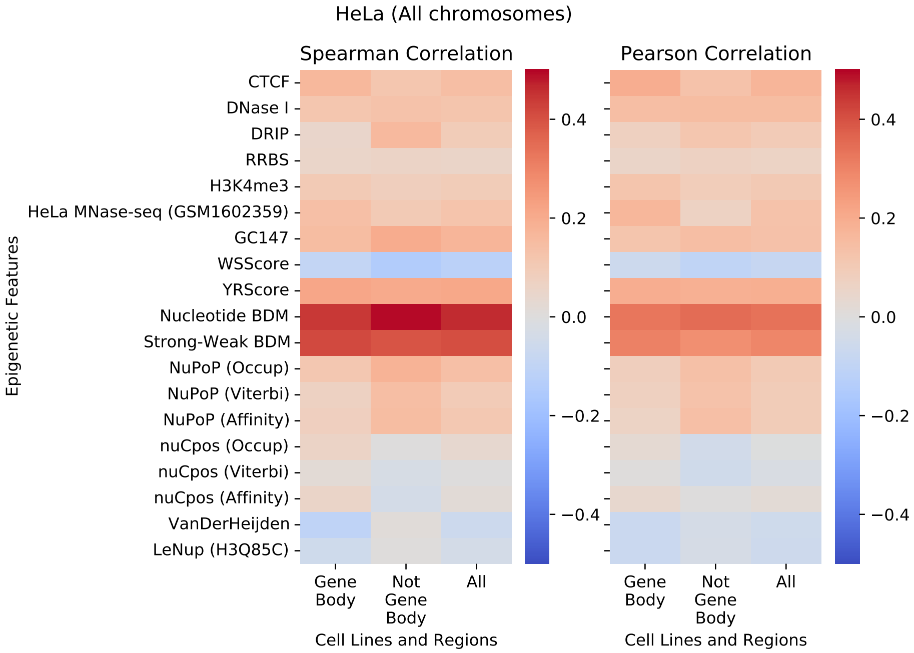

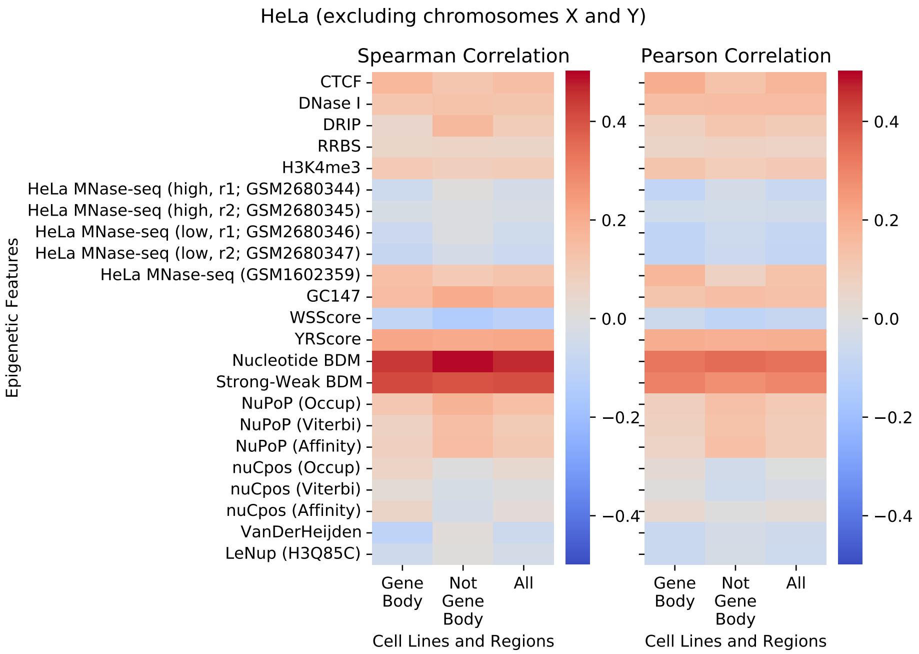

Supplementary Figure 2: (Top) Heatmaps showing Spearman (top left) and Pearson (top right) correlations between SpCas9 off-target cleavage activities and 19 epigenetic features, namely 13 computed nucleosome organization-related scores, 5 experimental epigenetic scores (bolded) and one HeLa MNase-seq score for HeLa-only nucleosome organization-related score-augmented off-target cleavage activity data. (Bottom) Heatmaps showing Spearman (bottom left) and Pearson (bottom right) correlations between SpCas9 off-target cleavage activities and 23 epigenetic features, namely 13 computed nucleosome organization-related scores, 5 experimental epigenetic scores (bolded) and 5 HeLa MNase-seq scores for HeLa-only nucleosome organization-related score-augmented off-target cleavage activity data in chromosomes 1-22.

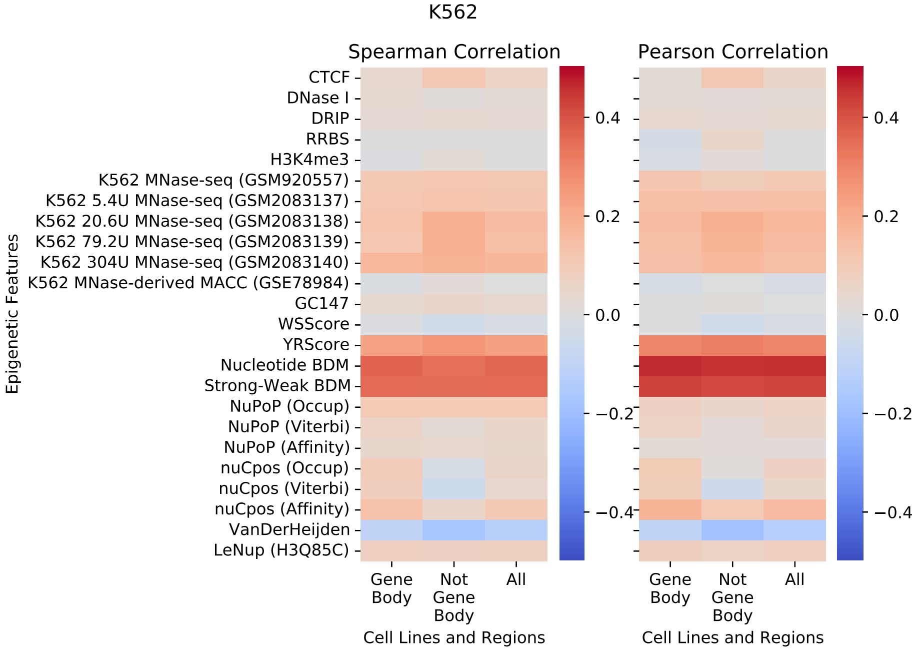

Supplementary Figure 3: Heatmaps showing Spearman (left) and Pearson (right) correlations between SpCas9 off-target cleavage activities and 24 epigenetic features, namely 13 computed nucleosome organization-related scores, 5 experimental epigenetic scores (bolded) and 6 K562 MNase-seq score for K562-only nucleosome organization-related score-augmented off-target cleavage activity data.

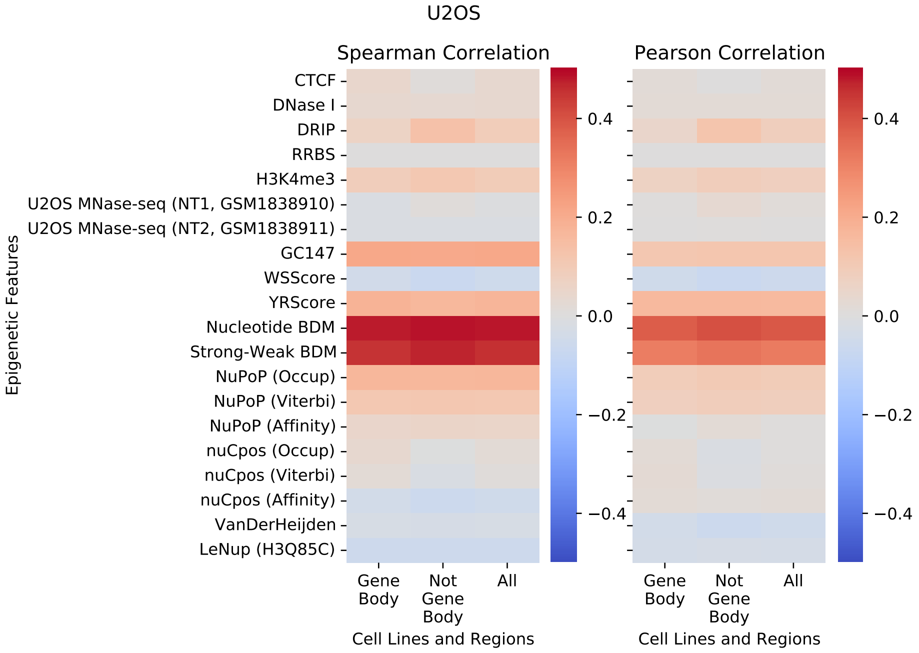

Supplementary Figure 4: Heatmaps showing Spearman (left) and Pearson (right) correlations between SpCas9 off-target cleavage activities and 20 epigenetic features, namely 13 computed nucleosome organization-related scores, 5 experimental epigenetic scores (bolded) and 2 U2OS MNase-seq score for U2OS-only nucleosome organization-related score-augmented off-target cleavage activity data.

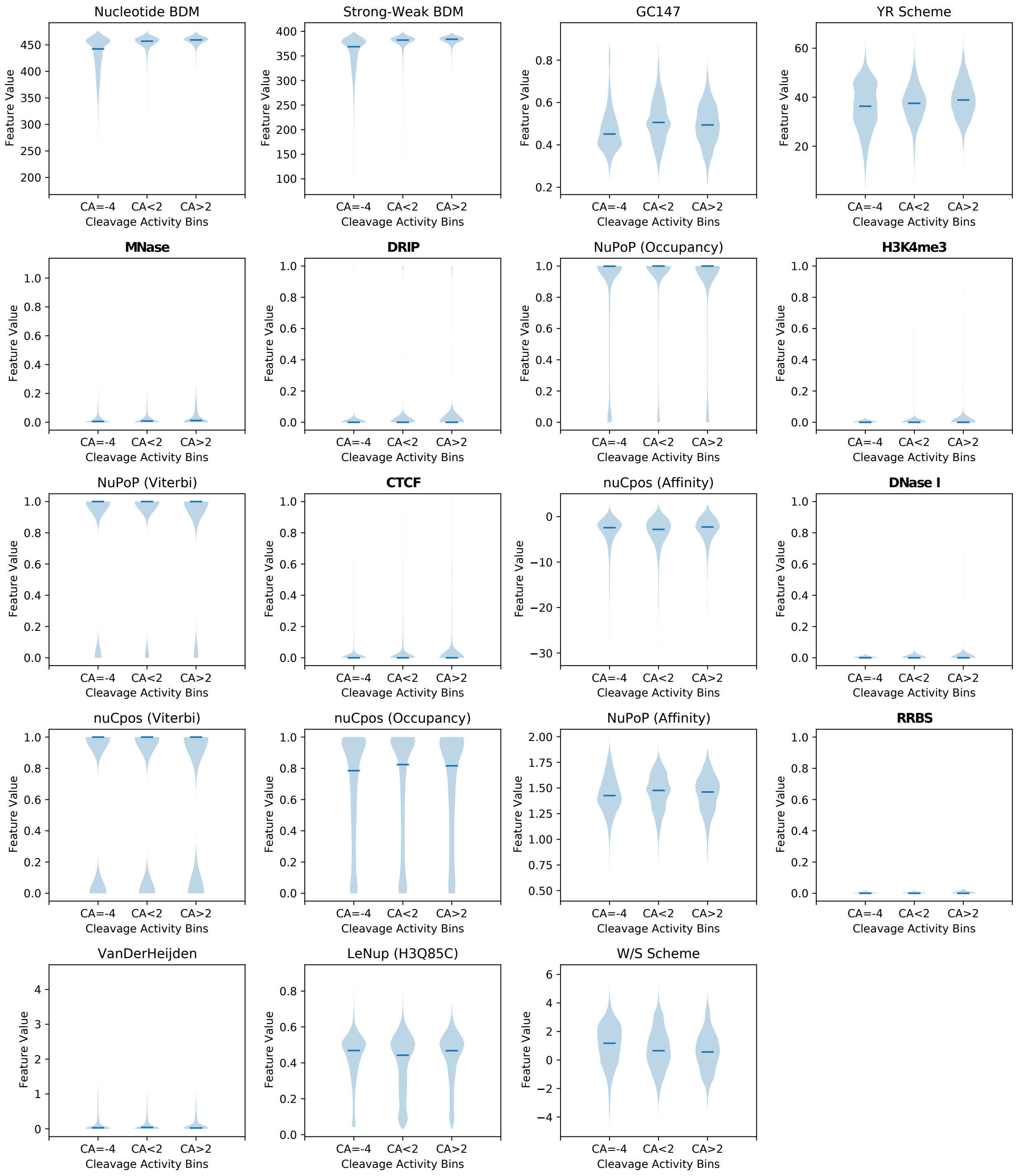

Supplementary Figure 5: Violin plots for all nucleosome organization-related features, with the features sorted decreasing Pearman correlation with CRISPR-Cas9 activity values and the experimental epigenetic features CTCF, DNase I, DRIP, H3K4me3, MNase and RRBS highlighted in bold. Cleavage activities (CA) are separated into three bins, namely CA $= -4$, CA < 2 and CA > 2.

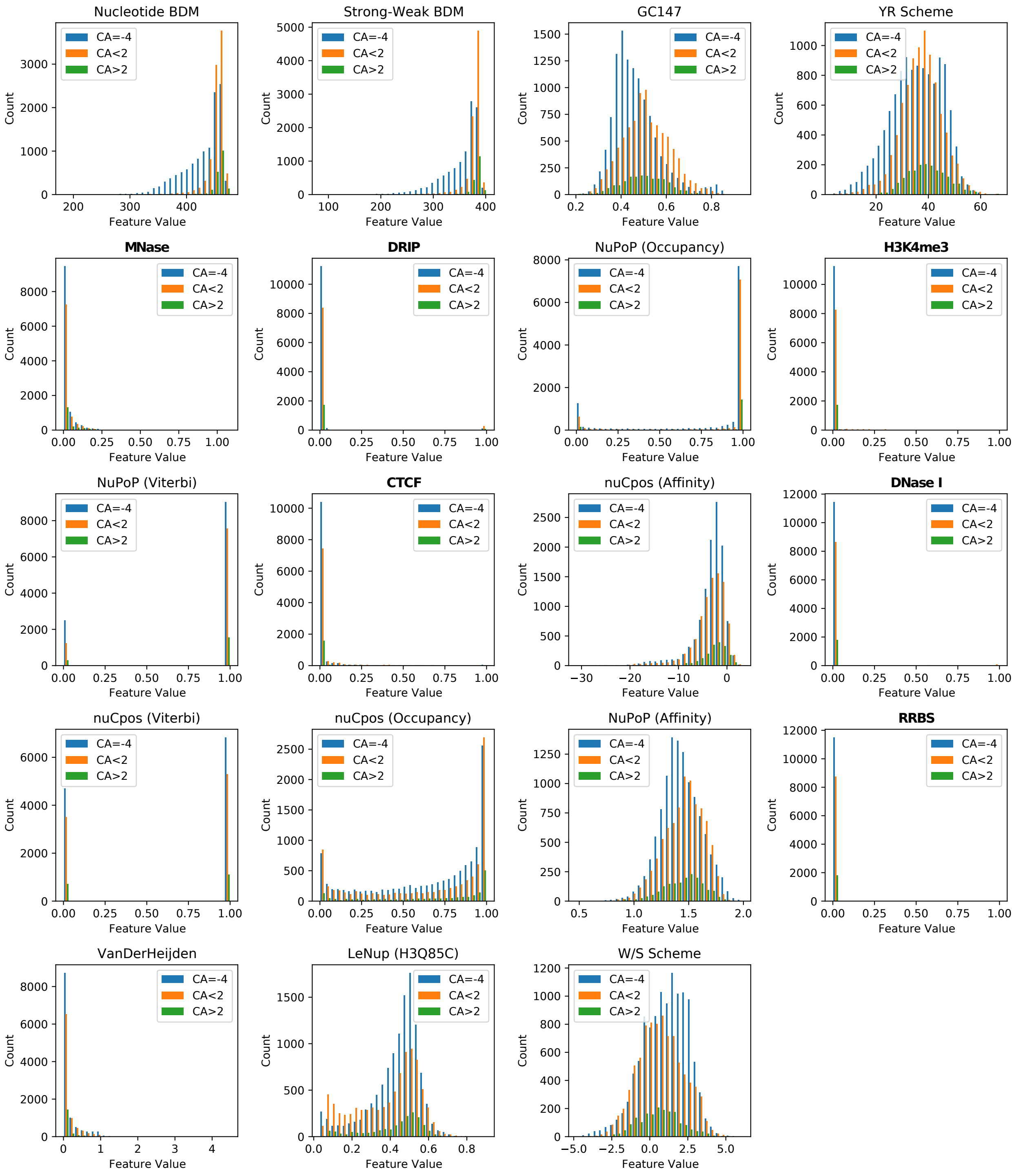

Supplementary Figure 6: Distribution plots for all nucleosome organization-related features, with the features sorted decreasing Spearman correlation with CRISPR-Cas9 activity values and the experimental epigenetic features CTCF, DNase I, DRIP, H3K4me3, MNase and RRBS highlighted in bold. Cleavage activities (CA) are separated into three bins, namely $CA = -4$, $CA < 2$ and $CA > 2$, which are colored blue, orange and green, respectively, in the plots.

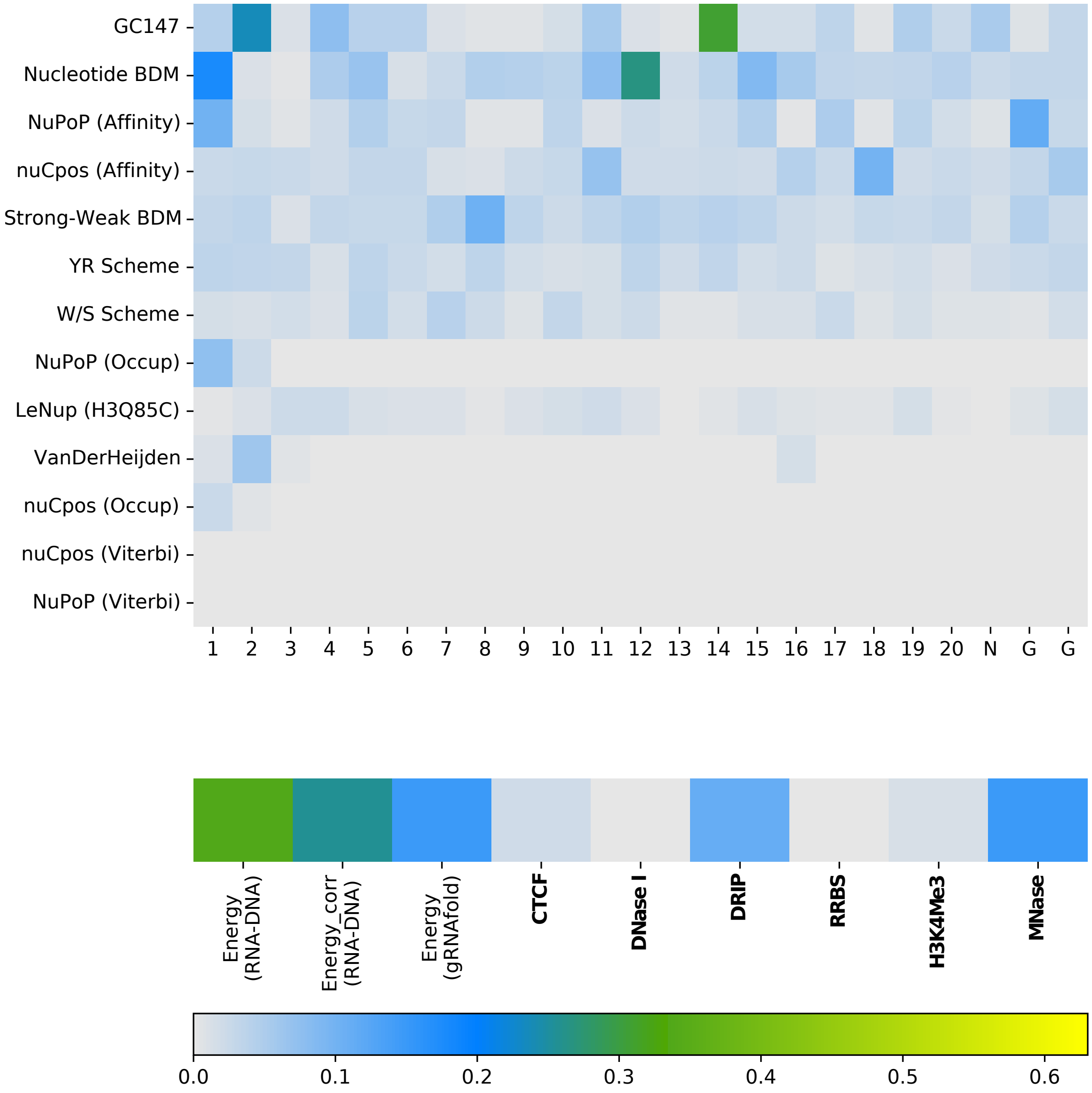

Supplementary Figure 7: Heatmap showing the mean absolute value of the SHAP values for the trained extreme gradient boosted (XGBoost) tree's base pair-resolved input features, which consist of the three CRISPRspec-derived energy terms $E_{\text{RNA-DNA}}$, $E^{\text{corr}}_{\text{RNA-DNA}}$ and E_{gRNAfold}, the six experimental epigenetic scores CTCF, DNase I, DRIP, RRBS, H3K4me3 and MNase (bolded), and the computed nucleosome organization-related scores GC147 [3], W/S scheme, YR scheme [4, 5], Strong-Weak BDM, Nucleotide BDM [6, 7], NuPoP (Occupancy), NuPoP (Affinity), NuPoP (Viterbi) [8], nuCpos (Occupancy), nuCpos (Affinity), nuCpos (Viterbi) [9], VanDerHeijden [10] and LeNup (H3Q85C) [11], with the computed scores sorted by decreasing SHAP value importance as shown in Figure 3.

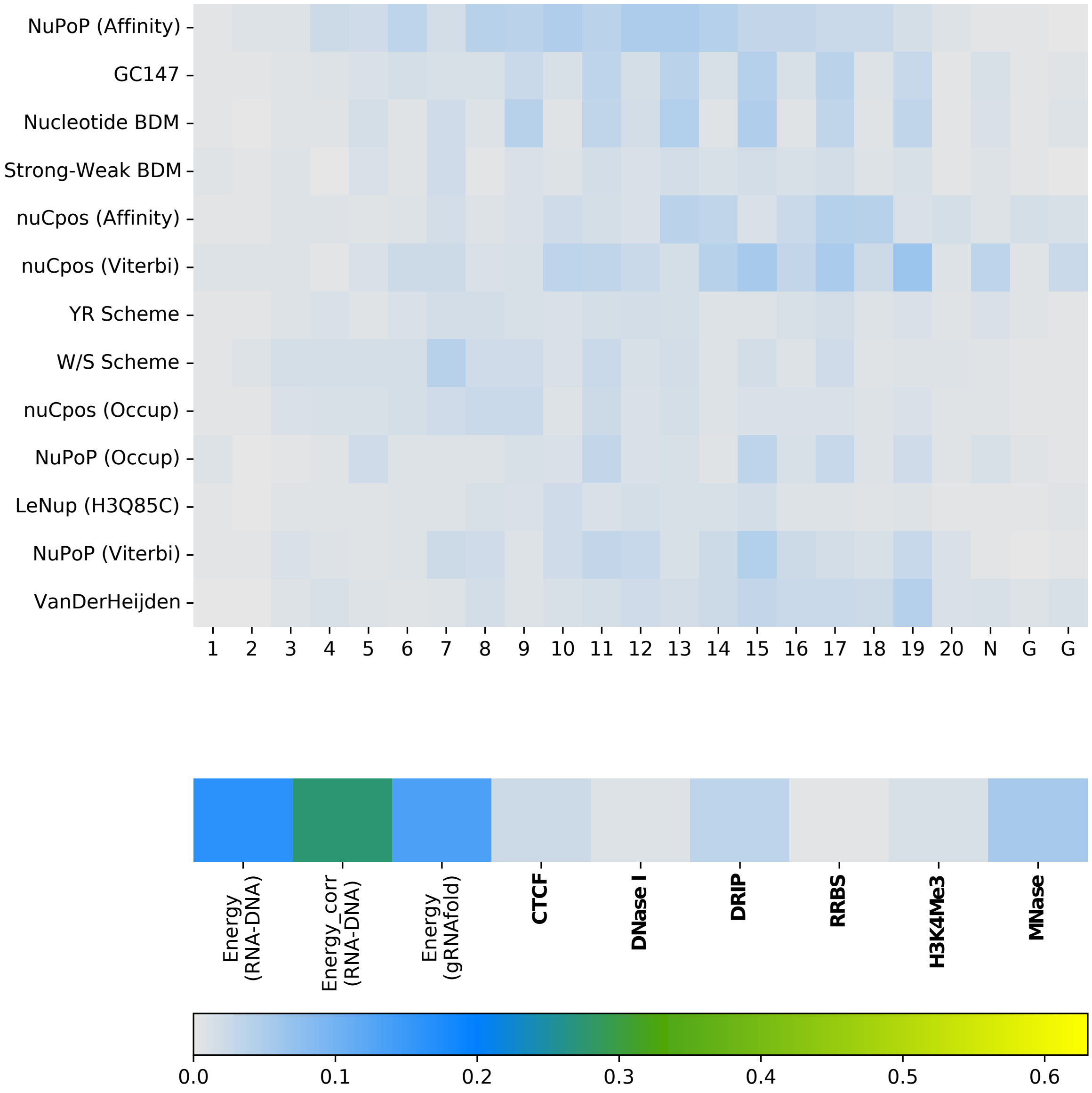

Supplementary Figure 8: Heatmap showing the mean absolute value of the SHAP values for the trained convolutional neural network's (CNN) base pair-resolved input features, which consist of the three CRISPRspec-derived energy terms $E_{\text{RNA-DNA}}$, $E^{\text{corr}}_{\text{RNA-DNA}}$ and E_{gRNAfold}, the four experimental epigenetic scores CTCF, DNase I, DRIP, RRBS, H3K4me3 and MNase (bolded), and the computed nucleosome organization-related scores GC147 [3], W/S scheme, YR scheme [4, 5], Strong-Weak BDM, Nucleotide BDM [6, 7], NuPoP (Occupancy), NuPoP (Affinity), NuPoP (Viterbi) [8], nuCpos (Occupancy), nuCpos (Affinity), nuCpos (Viterbi) [9], VanDerHeijden [10] and LeNup (H3Q85C) [11], with the computed scores sorted by decreasing SHAP value importance as shown in Figure 4.

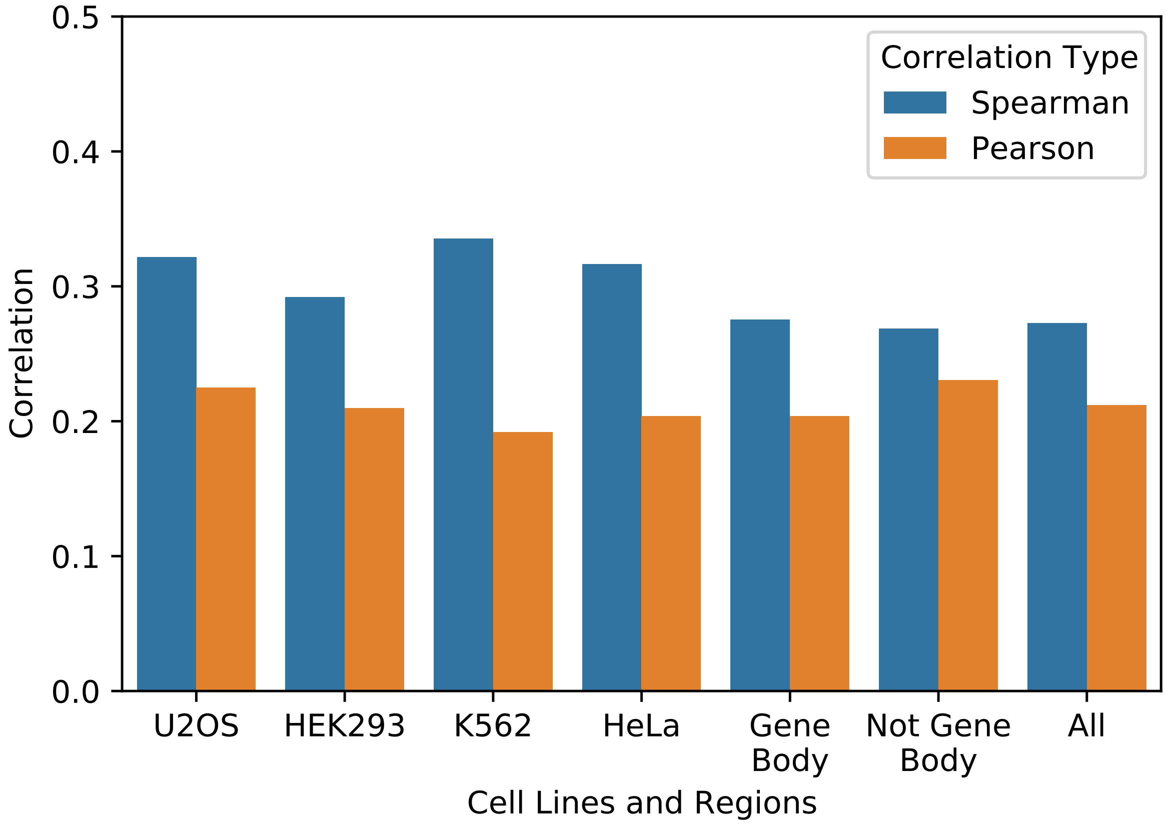

Supplementary Figure 9: Spearman and Pearson Correlations between NuPoP (Affinity) and Nucleotide BDM across different cell lines (U2OS, HEK293, K562, HeLa) and regions (Gene Body, Not Gene Body) for the dataset used in Figure 1.

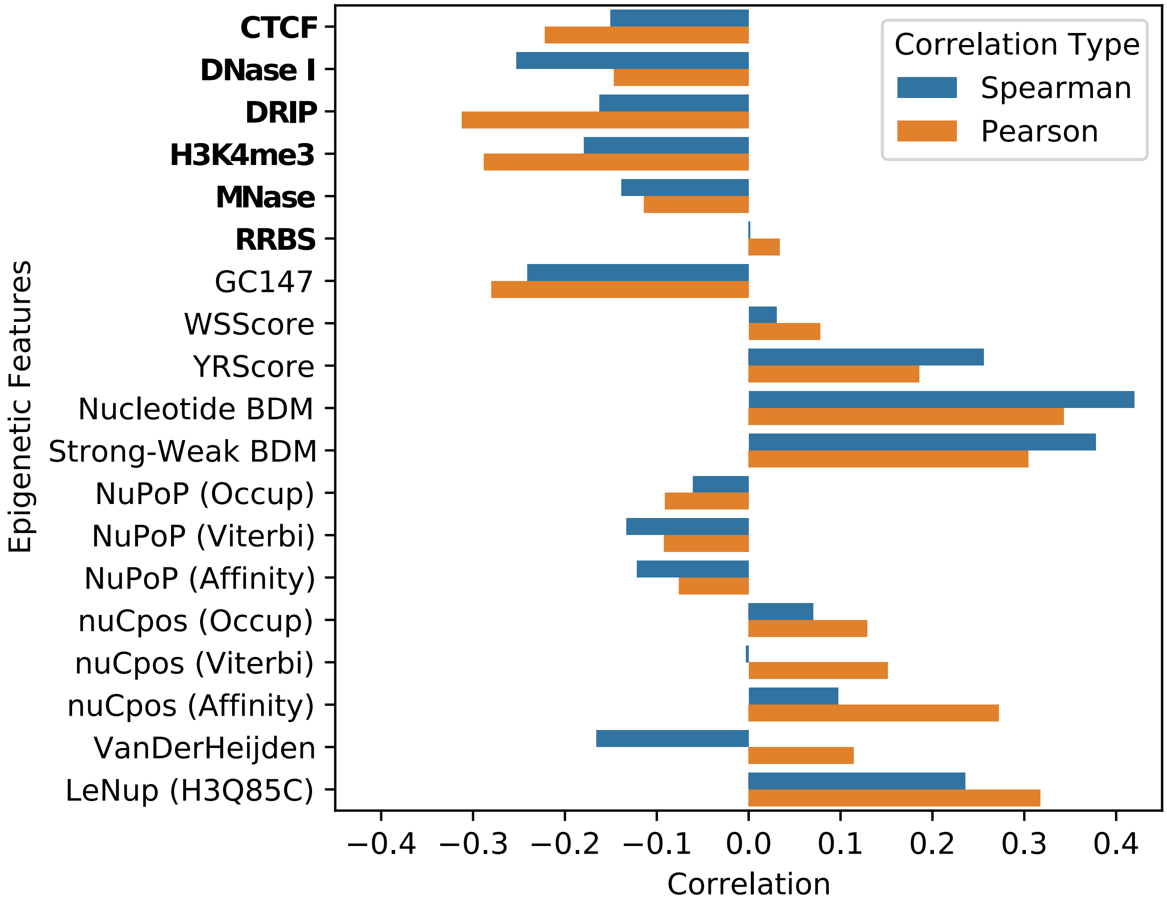

Supplementary Figure 10: Bar plot showing Spearman and Pearson correlations between 19 epigenetic features and SpCas9 on-target cleavage activities for all cell lines that contribute more than 1% to the crisprSQL dataset. The 19 epigenetic features consists of six experimental epigenetic features (bolded) and 13 nucleosome organization-related scores.

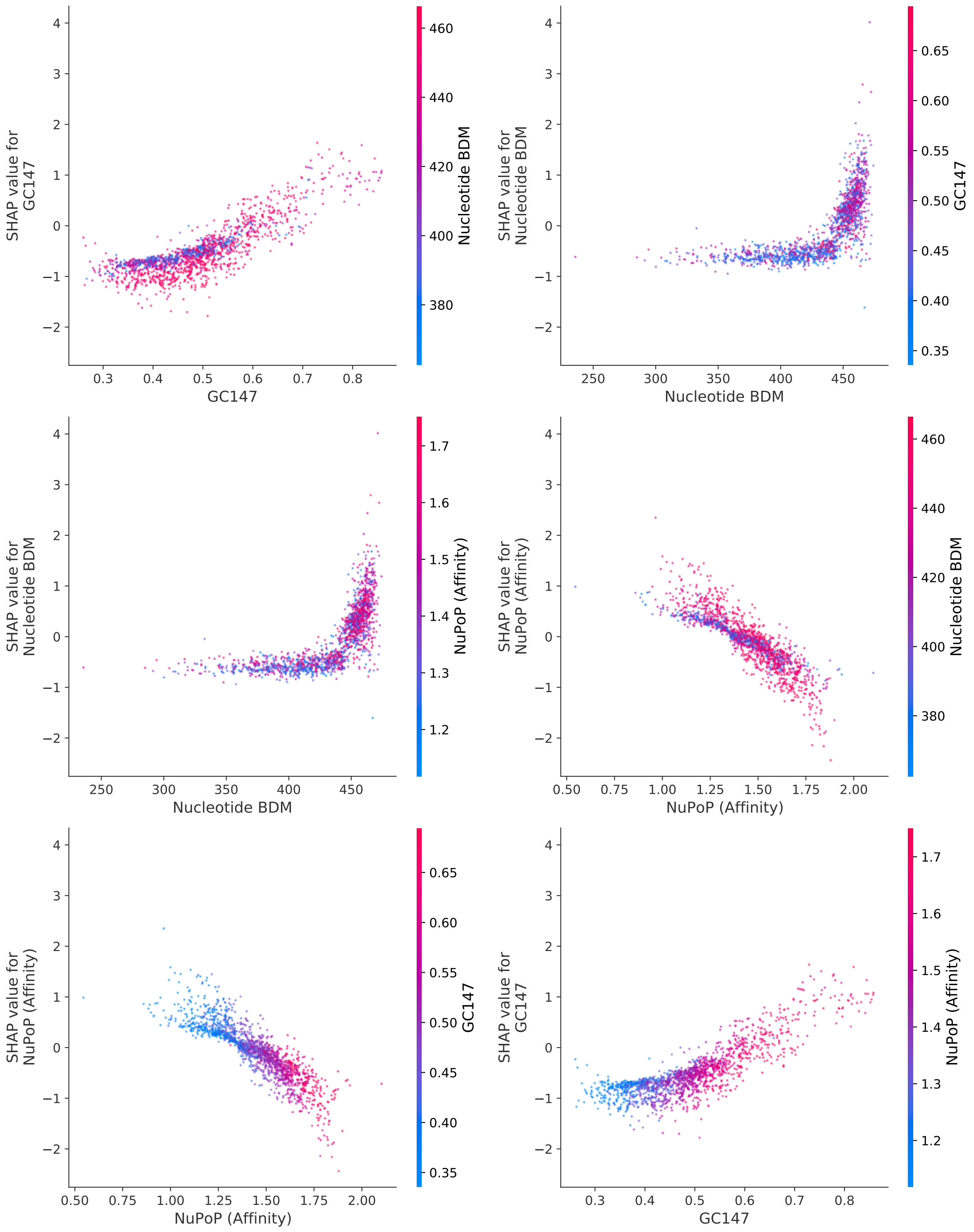

Supplementary Figure 11: SHAP dependency plots for GC147, Nucleotide BDM and NuPoP (Affinity) for XGBoost model.

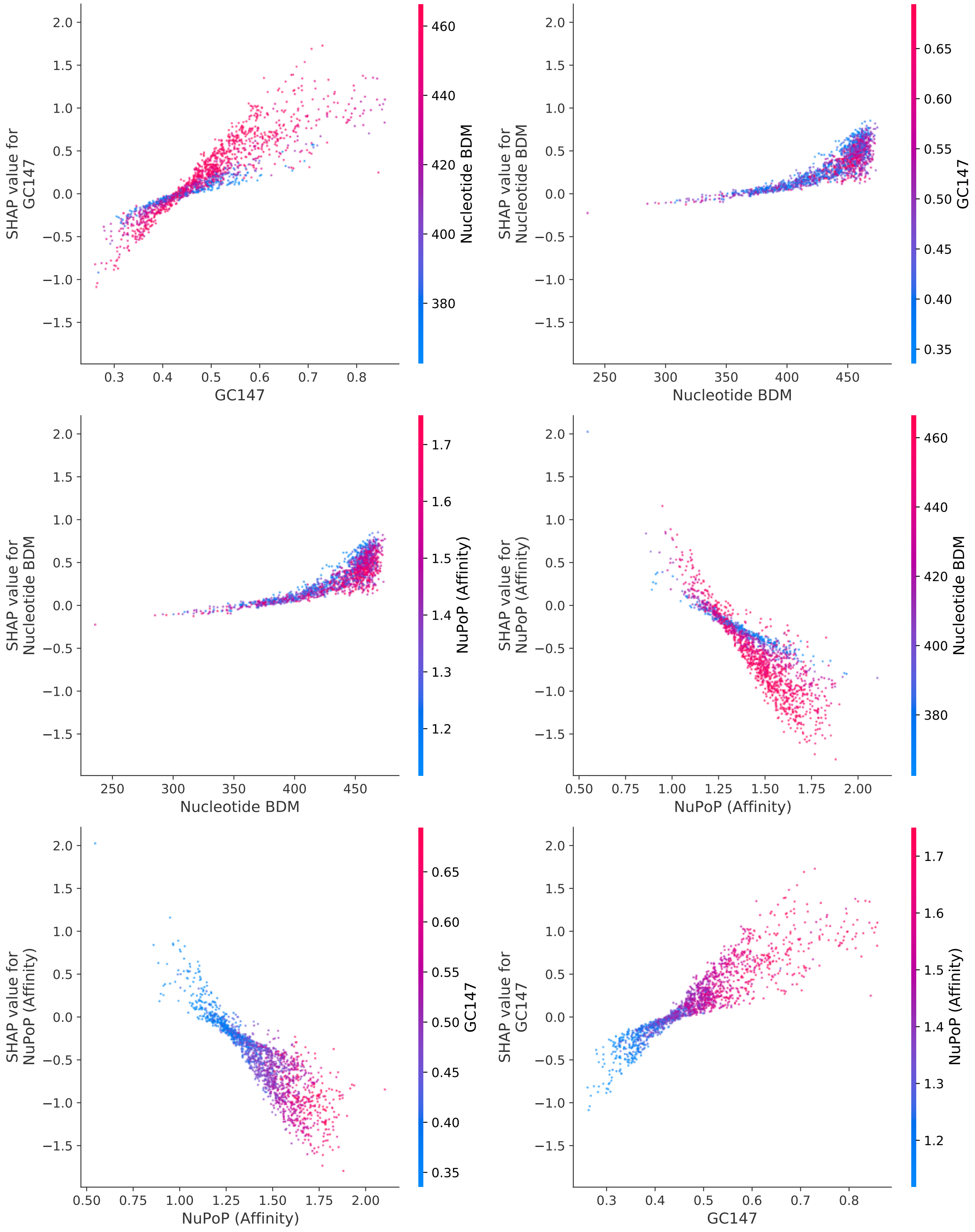

Supplementary Figure 12: SHAP dependency plots for GC147, Nucleotide BDM and NuPoP (Affinity) for CNN model.

2 Nucleosome Organization-Related Tools

This section provides additional background on some of the nucleosome organization-related tools used in this study.

2.1 GC Content/GC147

Mathematically, the GC content of a 147bp nucleosomal sequence s is defined by

$$GC147(s) = \frac{1}{147}\sum_{i=0}^{147} \mathbb{1}_{s_i \in \{G,C\}}.$$

GC content significantly correlates with *in vitro* nucleosome occupancy in budding yeast [12, 3]. Specifically, it was shown that GC content was the dominant feature in a linear model of nucleosome occupancy based on GC content and 14 other DNA sequence-related input features [3]. Nonetheless, GC content is not sufficient for *in vitro* nucleosome occupancy prediction, since it is not indicative of nucleosome occupancy levels in GC-rich regions *in vitro* [13].

2.2 W/S and YR Schemes

The W/S scheme is based on the well-established DNA sequence pattern where weak-weak (WW) and strong-strong (SS) dinucleotides are periodically located on the histone octamer-facing minor and major grooves, respectively. Mathematically, W/S scheme is defined as

$$\begin{aligned}\text{WSScore}(s) = & \sum_{s \in \text{minor sites}} C(WW, s) \\ & + \sum_{s \in \text{major sites}} C(SS, s) \\ & - \sum_{s \in \text{minor sites}} C(SS, s) \\ & - \sum_{s \in \text{major sites}} C(WW, s)\end{aligned}$$

where $W \in \{A, T\}$, $S \in \{C, G\}$, minor sites $= \{s \in \mathbb{Z} | -6 \leq s \leq 6\}$, major sites $= \{s + 0.5 | s \in \mathbb{Z}, -7 \leq s \leq 6\}$ and $C(\texttt{type}, s)$ denotes the number of `type` nucleotides in superhelical location (SHL) s.

The YR scheme is based on a weighted sum of GC, YR, YYRR and RYRY counts in the different sites. Further detailed descriptions on how the YR scheme predicts translational positioning can be found in [4] and [5].

2.3 Van Der Heijden Algorithm

Based on the dinucleotide wedge model [14], the likelihood ratio for each base pair position is given by

$$P_{\text{nuc}}(S) = 4^{|S|-1} \prod_{s=0}^{|S|-1} P_{\text{dinuc}}(s, S[s, s+1])$$

where S is the sequence with $|S| \approx 147$ centered on the base position. In addition, the position-dependent dinucleotide probabilities are defined by

$$P_{\text{dinuc}}(s, d) = \begin{cases} 0.25 + B\sin(\frac{2\pi s}{p}) & \text{if } d \in \{AA, TA, TT\} \\ 0.25 + \frac{B}{3}\sin(\frac{2\pi s}{p}) & \text{if } d \in \{GA, GG, GT\} \\ 0.25 - B\sin(\frac{2\pi s}{p}) & \text{if } d \in \{GC, TC, TG\} \\ 0.25 - \frac{B}{3}\sin(\frac{2\pi s}{p}) & \text{if } d \in \{AC, AG, AT\} \\ 0.25 & \text{otherwise} \end{cases}$$

where B and p are the amplitude and period of the dinucleotide frequencies, respectively. Using the likelihood ratios, an energy landscape can be derived. We can then apply the algorithm required for solving Percus's equation [15] in order to generate the nucleosome positioning scores. Nucleosome occupancy values can then be obtained by

applying a convolution operation with a 147bp uniform filter. To determine the algorithm's hyperparameters, a Levenberg–Marquadt routine [16] can be used for fitting periodicity p and chemical potential μ to experimental data. In particular, μ is a hyperparameter used when computing the solution to Percus's equation. More details on VanDerHeijden can be found in [10].

2.4 Block Decomposition Method-based Measures

Block Decomposition Method (BDM) is a training-free method for approximating the algorithmic complexity of sequences. Mathematically, BDM is founded on the Coding theorem method [17, 18], which relates algorithmic (Kolmogorov-Chaitin) complexity [19] with algorithmic probability [20]. Specifically, BDM approximates algorithmic complexity and Shannon entropy for short and long sequences, respectively [6]. Since DNA sequences can easily be represented as a string, BDM scores can readily be computed for DNA sequences.

2.5 NuPoP

NuPoP uses a dHMM and a Hidden Markov Model (HMM) for modelling 147bp nucleosomal and linker DNA sequences, respectively. Training data for both models consist of yeast nucleosomal and non-nucleosomal sequences derived from MNase-seq. Both models are used for computing log likelihood ratios, which can be seen as histone binding affinity (HBA) scores. Computationally, the HBA score at position i is given by $\log \frac{P_N(S_i)}{G_L(S_i)}$ where S_i is the 147bp sequence centered at position i. P_N and G_L indicate the probability that the S_i is a nucleosomal and linker sequence, respectively. Since linker sequences cannot be too long, NuPoP sets a maximum linker sequence length to 500bp for the dHMM. Using the HBA scores, the forward and backward algorithms can then be used for computing the nucleosome occupancy scores. A Viterbi score can also be computed, which predicts whether a specified nucleotide is located in nucleosomal or linker DNA. More details on the algorithm can be found in [8].

References

[1] Paszke, A. *et al.* Pytorch: An imperative style, high-performance deep learning library. In *Advances in Neural Information Processing Systems 32*, 8024–8035 (Curran Associates, Inc., 2019). URL `http://papers.neurips.cc/paper/9015-pytorch-an-imperative-style-high-performance-deep-learning-library.pdf`.

[2] Maas, A. L., Hannun, A. Y. & Ng, A. Y. Rectifier nonlinearities improve neural network acoustic models. In *in ICML Workshop on Deep Learning for Audio, Speech and Language Processing* (2013).

[3] Tillo, D. & Hughes, T. R. G+c content dominates intrinsic nucleosome occupancy. *BMC Bioinformatics* **10** (2009).

[4] Cui, F. & Zhurkin, V. B. Structure-based analysis of DNA sequence patterns guiding nucleosome positioningin vitro. *Journal of Biomolecular Structure and Dynamics* **27**, 821–841 (2010).

[5] Alharbi, B. A., Alshammari, T. H., Felton, N. L., Zhurkin, V. B. & Cui, F. nuMap: A web platform for accurate prediction of nucleosome positioning. *Genomics, Proteomics & Bioinformatics* **12**, 249–253 (2014).

[6] Zenil, H., Hernández-Orozco, S., Kiani, N. A., Soler-Toscano, F. & Rueda-Toicen, A. A decomposition method for global evaluation of shannon entropy and local estimations of algorithmic complexity (2016). `1609.00110`.

[7] Zenil, H. & Minary, P. Training-free measures based on algorithmic probability identify high nucleosome occupancy in DNA sequences. *Nucleic Acids Research* **47**, e129–e129 (2019).

[8] Xi, L. *et al.* Predicting nucleosome positioning using a duration hidden markov model. *BMC Bioinformatics* **11**, 346 (2010).

[9] Kato, H., Shimizu, M. & Urano, T. Chemical map–based prediction of nucleosome positioning using the bioconductor package nucpos. *bioRxiv* (2019).

[10] van der Heijden, T., van Vugt, J. J., Logie, C. & van Noort, J. Sequence-based prediction of single nucleosome positioning and genome-wide nucleosome occupancy. *Proceedings of the National Academy of Sciences* **109**, E2514–E2522 (2012).

[11] Zhang, J., Peng, W. & Wang, L. LeNup: learning nucleosome positioning from DNA sequences with improved convolutional neural networks. *Bioinformatics* **34**, 1705–1712 (2018).

[12] Segal, E. *et al.* A genomic code for nucleosome positioning. *Nature* **442**, 772–778 (2006).

[13] Minary, P. & Levitt, M. Training-free atomistic prediction of nucleosome occupancy. *Proceedings of the National Academy of Sciences* **111**, 6293–6298 (2014).

[14] Satchwell, S. C., Drew, H. R. & Travers, A. A. Sequence periodicities in chicken nucleosome core dna. *Journal of Molecular Biology* **191**, 659 – 675 (1986).

[15] Vanderlick, T. K., Scriven, L. E. & Davis, H. T. Solution of Percus's equation for the density of hard rods in an external field. *Phys Rev A Gen Phys* **34**, 5130–5131 (1986).

[16] Levenberg, K. A method for the solution of certain non-linear problems in least squares. *The Quarterly of Applied Mathematics* 164–168 (1944).

[17] Delahaye, J.-P. & Zenil, H. Numerical evaluation of algorithmic complexity for short strings: A glance into the innermost structure of randomness. *Applied Mathematics and Computation* **219**, 63–77 (2012). Towards a Computational Interpretation of Physical Theories.

[18] Soler-Toscano, F., Zenil, H., Delahaye, J.-P. & Gauvrit, N. Calculating kolmogorov complexity from the output frequency distributions of small turing machines. *PLOS ONE* **9**, 1–18 (2014).

[19] Kolmogorov, A. N. On tables of random numbers (reprinted from "sankhya: The indian journal of statistics", series a, vol. 25 part 4, 1963). *Theor. Comput. Sci.* **207**, 387–395 (1998).

[20] Solomonoff, R. A formal theory of inductive inference. parts i and ii. *Information and Control* **7**, 1–22 and 224–254 (1964).
